# L-SIGN is a receptor on liver sinusoidal endothelial cells for SARS-CoV-2 virus

**DOI:** 10.1172/jci.insight.148999

**Published:** 2021-07-22

**Authors:** Yuji Kondo, Jason L. Larabee, Liang Gao, Huiping Shi, Bojing Shao, Christopher M. Hoover, J. Michael McDaniel, Yen-Chun Ho, Robert Silasi-Mansat, Stephanie A. Archer-Hartmann, Parastoo Azadi, R. Sathish Srinivasan, Alireza R. Rezaie, Alain Borczuk, Jeffrey C. Laurence, Florea Lupu, Jasimuddin Ahamed, Rodger P. McEver, James F. Papin, Zhongxin Yu, Lijun Xia

**Affiliations:** 1 Cardiovascular Biology Research Program, Oklahoma Medical Research Foundation, Oklahoma City, Oklahoma, USA.; 2 Department of Microbiology and Immunology and; 3 Department of Biochemistry and Molecular Biology, University of Oklahoma Health Sciences Center, Oklahoma City, Oklahoma, USA.; 4 Complex Carbohydrate Research Center, University of Georgia, Athens, Georgia, USA.; 5 Department of Pathology and Laboratory Medicine and; 6 Division of Hematology and Medical Oncology, Weill Cornell Medicine, New York, New York, USA.; 7 Department of Pathology and; 8 Department of Physiology, University of Oklahoma Health Sciences Center, Oklahoma City, Oklahoma, USA.

**Keywords:** Cell Biology, Vascular Biology, Coagulation, Endothelial cells

## Abstract

Coronavirus disease 2019 (COVID-19), caused by severe acute respiratory syndrome coronavirus 2 (SARS-CoV-2), remains a pandemic. Severe disease is associated with dysfunction of multiple organs, but some infected cells do not express ACE2, the canonical entry receptor for SARS-CoV-2. Here, we report that the C-type lectin receptor L-SIGN interacted in a Ca^2+^-dependent manner with high-mannose–type N-glycans on the SARS-CoV-2 spike protein. We found that L-SIGN was highly expressed on human liver sinusoidal endothelial cells (LSECs) and lymph node lymphatic endothelial cells but not on blood endothelial cells. Using high-resolution confocal microscopy imaging, we detected SARS-CoV-2 viral proteins within the LSECs from liver autopsy samples from patients with COVID-19. We found that both pseudo-typed virus enveloped with SARS-CoV-2 spike protein and authentic SARS-CoV-2 virus infected L-SIGN–expressing cells relative to control cells. Moreover, blocking L-SIGN function reduced CoV-2–type infection. These results indicate that L-SIGN is a receptor for SARS-CoV-2 infection. LSECs are major sources of the clotting factors vWF and factor VIII (FVIII). LSECs from liver autopsy samples from patients with COVID-19 expressed substantially higher levels of vWF and FVIII than LSECs from uninfected liver samples. Our data demonstrate that L-SIGN is an endothelial cell receptor for SARS-CoV-2 that may contribute to COVID-19–associated coagulopathy.

## Graphical abstract

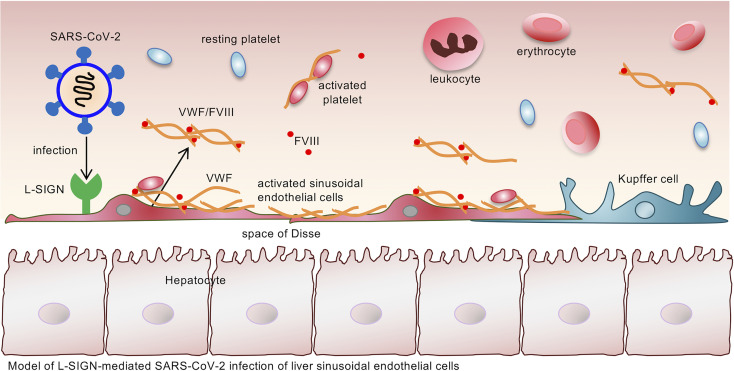

## Introduction

Severe acute respiratory syndrome coronavirus 2 (SARS-CoV-2) infection causes coronavirus disease 2019 (COVID-19). Despite an unprecedented worldwide effort, COVID-19 remains a pandemic, with global deaths approaching 3.1 million (1). While the majority of the infected individuals are asymptomatic, symptomatic patients have a wide spectrum of clinical manifestations, ranging from viral pneumonia and acute respiratory distress syndrome to extrapulmonary injuries to the heart, kidney, liver, and other organs. Multiorgan involvement is an important risk factor for severity and mortality.

SARS-CoV-2 is an enveloped, positive-sense RNA member of the betacoronavirus genus known to infect humans. Angiotensin-converting enzyme 2 (ACE2) is the first documented receptor mediating the entry of SARS-CoV-2 into host cells (2, 3). ACE2 expression has been found on alveolar epithelial type II cells of the lung and on nasal epithelial cells, renal tubular cells, and intestinal epithelial cells (4), and this expression is thought to contribute to virus-mediated injury of these organs. Up to 71.4% of critically ill patients with COVID-19 have thrombosis and coagulopathy, which are often associated with poor prognosis (5, 6). Multiple studies, including a multicenter prospective cohort study, indicate that coagulopathy and thrombosis in patients with COVID-19 are characterized by increased plasma levels of D-dimer (fibrin degradation product), vWF, and coagulation factor VIII (FVIII; refs. 7, 8), which is thought to be caused by SARS-CoV-2–induced inflammatory responses or sepsis (2, 7, 8). Markers of endothelial activation, such as elevated levels of soluble P selectin, CD40L, and thrombomodulin, have been documented in the plasma of patients with severe COVID-19 (7). However, recent single-cell RNA-sequence studies have demonstrated little or no expression of ACE2 in vascular endothelial cells (4, 9). These findings have left unresolved whether direct SARS-CoV-2 infection of endothelial cells contributes to coagulopathy and thrombosis (5, 7, 8).

SARS-CoV-2 enters host cells through binding of its spike protein to the ACE2 receptor (2, 3). Like other viral spike proteins, such as those causing Ebola and Marburg hemorrhagic fever, the SARS-CoV-2 spike protein is highly glycosylated to form a “glycan shield” to evade host immune surveillance (10–12). Recently, several groups identified “complex type” and “high-mannose–type” N-linked glycans on the SARS-CoV-2 spike protein (10, 11, 13, 14), and it was suggested that several glycosylation sites contribute to masking of immunogenic epitopes from immune recognition, conformational change of spike protein, and interaction with ACE2 (13, 15–17).

SARS-CoV-2 is related to SARS-CoV (~80% sequence identity), which caused the epidemic of severe acute respiratory syndrome in 2003. Previous studies have shown that SARS-CoV, in addition to interacting with ACE2, binds to liver/lymph node–specific ICAM-3 grabbing nonintegrin (L-SIGN, also known as CD209L, CD299, or CLEC4M), which mediates virus entry into host cells (18–20). L-SIGN is a C-type lectin receptor that mediates cell adhesion or pathogen recognition by Ca^2+^-dependent binding to glycans. Whether SARS-CoV-2 uses L-SIGN to infect host cells is unknown. Here, we report that L-SIGN bound to SARS-CoV-2 spike protein in a high-mannose–type N-glycan– and Ca^2+^-dependent manner. L-SIGN was detected at high levels on liver sinusoidal endothelial cells (LSECs) and on lymphatic endothelial cells (LECs) of lymph nodes; however, in contrast to findings of prior reports (19), it was not detected on blood endothelial cells from human biopsy or autopsy samples. Authentic SARS-CoV-2 virus infected L-SIGN–expressing LSECs but not control cells. LSECs and LECs are major sources of procoagulant proteins, such as FVIII and vWF (21–23). We detected increased expression of vWF and FVIII in LSECs from liver samples from patients with COVID-19. This study demonstrates that L-SIGN is an endothelial cell receptor for SARS-CoV-2 and that interaction between SARS-CoV-2 and L-SIGN on LSECs may contribute to COVID-19–associated coagulopathy.

## Results

### The SARS-CoV-2 spike protein interacts with L-SIGN.

**Figure 1:**
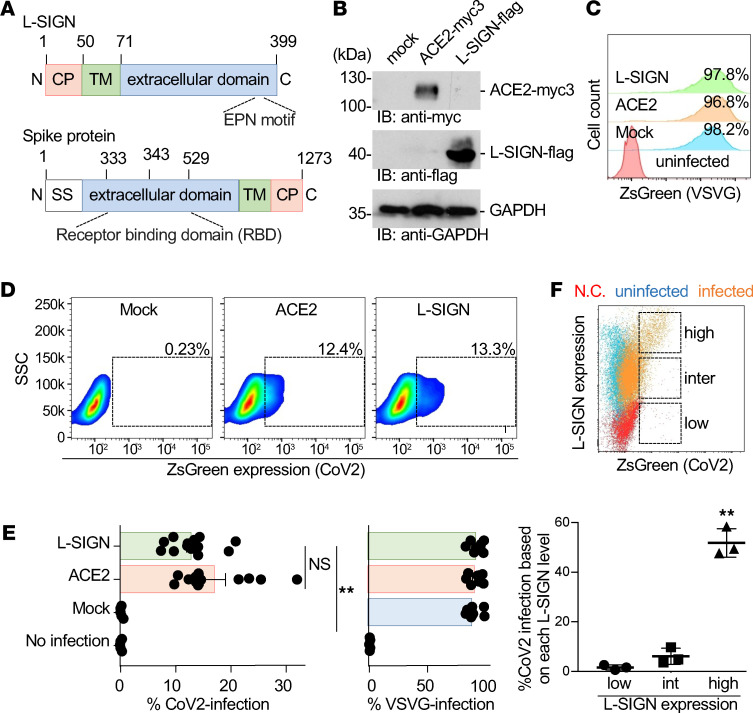
Binding of L-SIGN to the SARS-CoV-2 spike protein mediates viral entry. (**A**) Protein domain structures of SARS-CoV-2 spike protein and human L-SIGN (numbers represent amino acids). TM, transmembrane domain; CP, cytoplasmic domain; EPN, Glu-Pro-Asn motif; SS, signal sequence. (**B**) Protein expression of ACE2-myc3 and L-SIGN-flag in transfected HEK293T cells by immunoblotting. GAPDH was used as a loading control. (**C** and **D**) Representative flow cytometry plots of VSVG-type (histogram) and SARS-CoV-2–type (density plot) pseudo-typed virus infection in response to mock-transfected, ACE2-myc3–transfected, and L-SIGN-flag–transfected HEK293T cells defined by ZsGreen expression. Uninfected or mock-transfected cells were used as negative control. Dashed boxes indicate infected cells. (**E**) Quantification of pseudo-typed virus infection in **C** and **D**. Each dot represents an individual experiment. (**F**) Representative overlaid flow cytometry plot of exogenous L-SIGN expression and CoV-2–type infection on L-SIGN-flag–transfected HEK293T cells (top) and quantification of the percentage of CoV-2 infection based on different L-SIGN expression levels (bottom). CoV-2 infectivity positively correlates with L-SIGN expression level. Percentages of infection were normalized with percentages of expression of L-SIGN in HEK293T cells. N.C., negative control; uninfected, L-SIGN–stained HEK293T cells; infected, L-SIGN–stained CoV-2–type–infected HEK293T cells; Inter, intermediate. Bars indicate the mean; error bars represent mean ± SEM. Significance was calculated using a 1-way ANOVA for multiple groups and a 2-tailed Student’s *t* test for comparing 2 groups: *P < 0.05; **P < 0.01. All experiments were repeated at least 3 times.

The extracellular domain of the SARS-CoV-2 spike protein has a receptor-binding domain (RBD; Figure 1A and refs. 10, 11). To examine if L-SIGN interacts with SARS-CoV-2, we generated lentivirus-based SARS-CoV-2 pseudo-typed virus (CoV-2–type virus), and established HEK293T cell lines expressing L-SIGN-flag or ACE2-myc3 as positive control (Figure 1B and ref. 2). The lentivirus-based CoV-2–type virus is widely used to measure ACE2-mediated viral entry. Infection can be monitored by expression of ZsGreen fluorescent protein after integration of ZsGreen DNA into the infected host genome (2). The control VSV-G pseudo-typed virus (VSVG-type virus) efficiently and nonspecifically infected HEK293T cells (Figure 1, C and E, and ref. 2). We found that CoV-2–type virus specifically infected cells expressing ACE2-myc3 or L-SIGN-flag (Figure 1, D and E). In addition, CoV-2–type virus preferentially infected cells with higher levels of L-SIGN expression (Figure 1F). The differences in infection efficiency between CoV-2–type and VSVG-type viruses are likely caused by differences between the viral glycoprotein interactions with specific cell surface receptors, of which both will differ for each virus (3, 24–26).

### L-SIGN binding to spike protein is high-mannose–type N-glycan and Ca^2+^ dependent.

**Figure 2:**
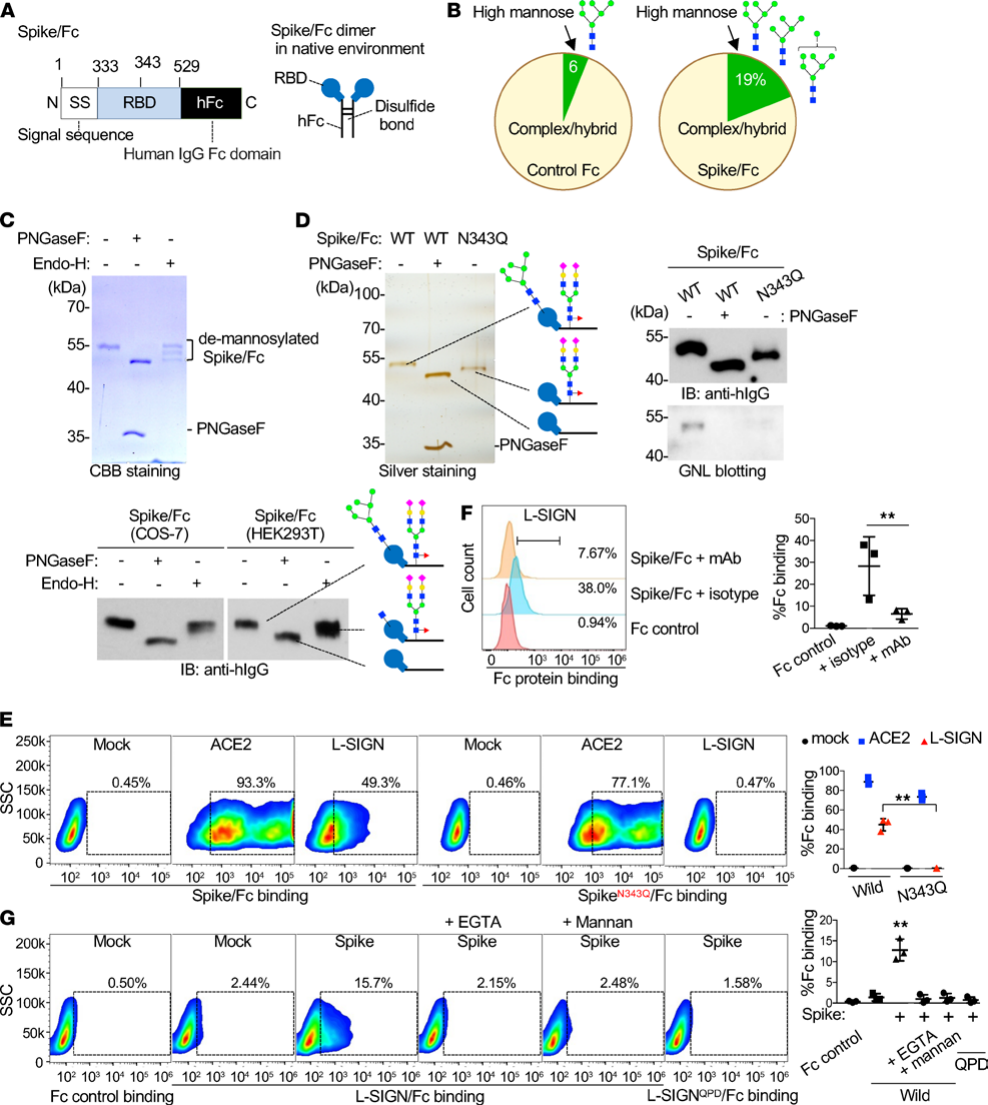
L-SIGN binds to a high-mannose–type N-glycan on the spike protein of SARS-CoV-2. (**A**) Protein domain structures of spike/Fc (left) (numbers represent amino acids). Spike/Fc forms dimer (right). RBD, receptor-binding domain; hFc, human IgG Fc domain. (**B**) Percentages of high-mannose–type versus complex/hybrid–type N-glycans in control Fc and spike/Fc proteins produced from COS-7 cells determined by MALDI-TOF MS. See also Supplemental Figure 1. (**C**) CBB staining of SDS-PAGE gels of COS-7 cell–derived spike/Fc under reducing conditions, with or without PNGaseF or Endo-H treatment (top). Immunoblotting of spike/Fc produced from COS-7 and HEK293T cells with or without PNGaseF or Endo-H treatment (bottom). (**D**) Silver staining of SDS-PAGE gel of COS-7 cell–derived purified Spike^N343Q^/Fc protein or of WT spike/Fc treated with or without PNGaseF. Immunoblots and GNL lectin blots of WT and Spike^N343Q^/Fc. The reduced size of Spike^N343Q^/Fc protein relative to that of WT indicates the loss of an N-glycan on the RBD. (**E**) Representative flow cytometry plots of spike^N343Q^/Fc binding to mock-, ACE2-myc3–, and L-SIGN-flag–transfected HEK293T cells (left) and quantification of the percentage of Fc protein binding (right). Dashed boxes indicate positive population. (**F**) Representative flow cytometry plot of spike/Fc binding to L-SIGN-flag–transfected HEK293T cells with the pretreatment of isotype IgG or anti–L-SIGN mAb (left) and quantification of the percentage of Fc protein binding (right). Isotype, isotype IgG pretreated; mAb, anti–L-SIGN mAb pretreated. (**G**) Representative flow cytometry plots of L-SIGN/Fc binding to spike protein–transfected HEK293T cells (left) and quantification of the percentage of Fc protein binding (right). Mannan (100 μg/ml), a mannose polymer, was used as a competitive inhibitor of L-SIGN/Fc. L-SIGN^QPD^/Fc is a carbohydrate recognition domain mutant of L-SIGN/Fc. EGTA (1 mM) was used as Ca^2+^ chelator. All experiments were repeated at least 3 times. For all analyses, *n =* 3. Bars indicate the mean; error bars represent mean ± SEM. Significance was calculated using a 1-way ANOVA for multiple groups: **P < 0.01.

The RBD sequence of the SARS-CoV-2 spike protein has an N-glycosylation site at Asn343 (N343; refs. 10, 11; Figure 1A; and Supplemental Figure 1A; supplemental material available online with this article; https://doi.org/10.1172/jci.insight.148999DS1). We generated a recombinant protein with the RBD (amino acids 333–529) of the spike protein fused to a human IgG2 Fc from African green monkey COS-7 cells (spike/Fc; Figure 2A). We analyzed the glycan structures of spike/Fc and found that high-mannose–type N-glycans were frequent (Figure 2B; Supplemental Figure 1, B and C; and ref. 27), a finding which was confirmed by their removal with the high-mannose–specific endoglycosidase H (Endo-H; Figure 2C). HEK293T cell–derived spike/Fc was also sensitive to Endo-H, indicating the presence of high-mannose moieties on spike/Fc expressed in both cell lines (Figure 2C). To determine whether a high-mannose–type N-glycan is the determinant of L-SIGN binding, we first generated an N-glycan–deficient spike/Fc, in which Asn343 was replaced with glutamine (Q) (Spike^N343Q^/Fc; Figure 2D). Mannose moieties on spike/Fc and their deficiency on Spike^N343Q^/Fc were demonstrated by mannose-specific Galanthus nivalis lectin (GNL) blotting (Figure 2D). We confirmed that spike/Fc bound to cells expressing ACE2-myc3 and L-SIGN-flag and that its binding to cells expressing L-SIGN-flag was inhibited by pretreatment of cells with anti–L-SIGN mAb (Figure 2, E and F). In comparison, Spike^N343Q^/Fc bound to cells expressing ACE2-myc3 but not to cells expressing L-SIGN-flag, indicating that L-SIGN recognizes an N-glycan structure on Asn343 (Figure 2E).

**Figure 3:**
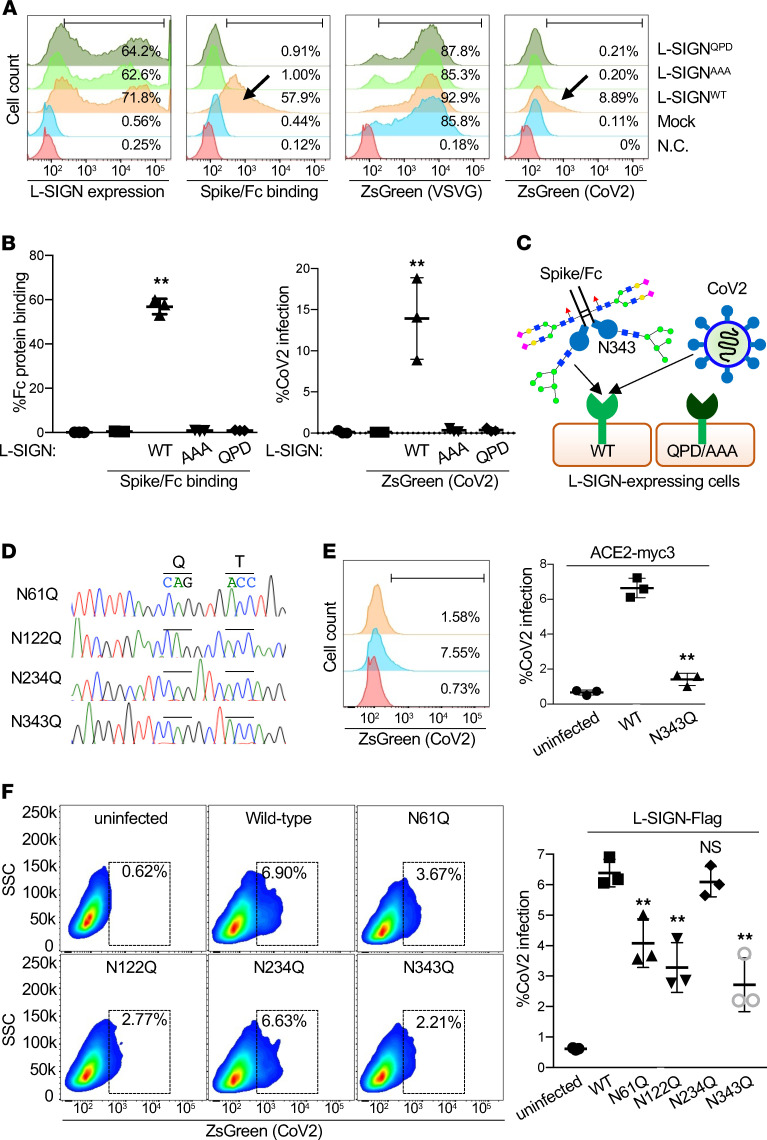
L-SIGN binding to spike protein depends on glycan recognition and calcium. (**A**) Representative flow cytometry histograms of surface expression of L-SIGN mutants, spike/Fc binding, and VSVG-type or SARS-CoV-2–type pseudo-typed viral infection in response to mock-transfected HEK293T cells (mock) or transfected HEK293T cells expressing the indicated L-SIGN construct. N.C. represents either isotype-matched, IgG-stained, Fc control or no viral infection. Arrows indicate positive staining or infection. (**B**) Quantification of the percentage of spike/Fc binding (left) and the percentage of CoV-2 infection (right) in **A**. (**C**) Cartoon of spike/Fc binding and CoV-2–type infection on L-SIGN expressing cells. QPD and AAA represent loss-of-function mutations of the carbohydrate recognition domain of L-SIGN. N343, Asn343. (**D**) DNA sequences of surrounding N-glycosylation sites (Asn-X-Thr), which have been mutated to (Gln-X-Thr) in each spike mutant. (**E**) Representative flow cytometry plot of N-glycosylation at Asn343-deficient CoV-2 infection in response to ACE2-myc3–transfected HEK293T cells (left) and quantification of CoV-2 infection (right). (**F**) Representative flow cytometry plots of each N-glycosylation–deficient CoV-2 infection in response to L-SIGN-flag–transfected HEK293T cells (left) and quantification of CoV-2 infection (right). Uninfected cells were used as negative control. Dashed boxes indicate infected cells. For all analyses, *n =* 3. Bars indicate the mean; error bars represent mean ± SEM. Significance was calculated using a 1-way ANOVA for multiple groups and a 2-tailed Student’s t test for comparing 2 groups: **P < 0.01.

L-SIGN has a Glu-Pro-Asn (EPN) motif in its extracellular domain that recognizes mannose moieties (Figure 1A and refs. 28, 29). To examine if the EPN motif of L-SIGN is involved in N-glycan recognition on spike/Fc, we substituted the EPN sequence with alanines (L-SIGN^AAA^) or with Gln-Pro-Asp (QPD), a galactose recognition motif (L-SIGN^QPD^; ref. 30). To investigate if a high-mannose–type N-glycan on spike/Fc is critical for L-SIGN binding, we expressed WT or mutant forms of the extracellular domain of L-SIGN (amino acids 78–399 amino acids) fused to the human IgG2 Fc domain (L-SIGN/Fc; Supplemental Figure 2A). L-SIGN/Fc but not L-SIGN^QPD^/Fc bound to spike protein–expressing HEK293T cells, and its binding was inhibited by Ca^2+^ chelator EGTA and by mannan, a β-mannose polymer that blocks interactions with high-mannose–type N-glycans (Figure 2G and ref. 31). Binding of spike/Fc and CoV-2 infection to ACE2-myc3–expressing cells were not inhibited by EGTA or mannan (Supplemental Figure 2, B and C). Spike/Fc binding and, importantly, CoV-2–type infection to cells expressing either L-SIGN mutants (L-SIGN^AAA^ or L-SIGN^QPD^) were abolished (Figure 3, A–C).

To further validate that high-mannose–type N-glycans are determinants of L-SIGN binding, we treated spike protein–expressing HEK293T cells with kifunensine, a small molecule that promotes expression of high-mannose–type N-glycans on the cell surface (Supplemental Figure 2D and ref. 32). Kifunensine treatment enhanced the binding of spike protein to either L-SIGN/Fc or GNL (Supplemental Figure 2, E–G). Although EGTA also chelate Mg^2+^ less efficiently, these data indicate that the EPN motif of L-SIGN recognizes a high-mannose–type N-glycan on spike protein in a Ca^2+^-dependent manner. SARS-CoV-2 spike protein has 22 potential N-glycosylation sites. Of these, Asn61, Asn122, Asn234, and Asn343 were reported to attach high-mannose–type N-glycans (Supplemental Figure 2H and refs. 10, 11, 13, 14). Mutagenesis study of these N-glycosylation sites revealed that Asn343 are important for ACE2-mediated SARS-CoV-2 infection (26). To further gain insight into high-mannose-dependent L-SIGN–mediated infection, we investigated the biological significance of N-glycan–deficient mutants at Asn61, Asn122, Asn234, and Asn343 in the SARS-CoV-2 spike protein (Figure 3D). Consistent with a previous report (26), mutation of Asn343 drastically reduced ACE2-mediated CoV-2 infection (Figure 3E). Mutation of Asn61, Asn122, or Asn343 significantly reduced L-SIGN–mediated CoV-2 infection (Figure 3F). Although Asn234 is reported to have high-mannose N-glycan, mutation of this site did not affect L-SIGN–mediated CoV-2 infectivity (Figure 3F and Supplemental Figure 2H).

### L-SIGN is highly expressed on LSECs in human biopsy or autopsy tissue samples.

**Figure 4:**
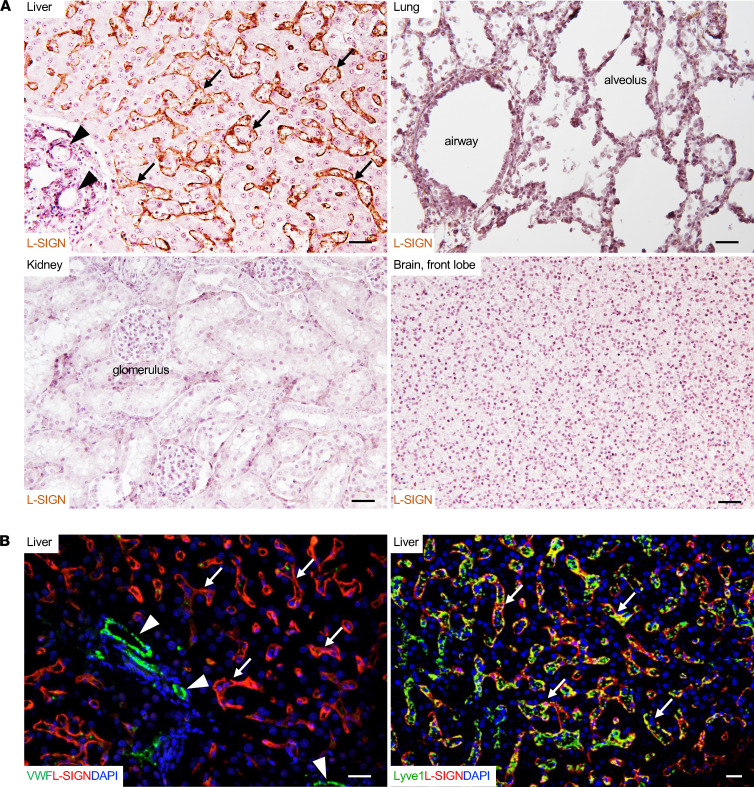
L-SIGN is expressed on LSECs in human biopsy or autopsy tissue samples. (**A**) Representative images of immunohistochemical staining of L-SIGN (ORIGENE, TA810067) in different normal human autopsy tissues. Arrows indicate liver sinusoids. Arrowheads indicate blood vessels. No L-SIGN was detected in blood vessels. Scale bar: 30 μm. (**B**) Representative confocal microscopy images of immunofluorescence staining of different normal human autopsy liver samples. Arrows indicate LSECs. Arrowheads indicate blood vessels. LSECs are vWF^+^Lyve1^+^L-SIGN^+^. Blood vessels are vWF^++^Lyve1^–^L-SIGN^–^. DAPI, nuclear staining. Scale bar: 20 μm. The results represent at least 3 experiments from biopsy or autopsy samples of 3 uninfected patients.

Only humans and great apes have genes that encode L-SIGN (33). To explore the biological function of the interaction between SARS-CoV-2 and L-SIGN, we first profiled the expression of L-SIGN in formalin-fixed paraffin-embedded normal human biopsy samples as well as autopsy tissue samples (34, 35). After antigen retrieval, L-SIGN was detected in LSECs and LECs in the lymph node with both immunohistochemical and immunofluorescence staining with 2 different antibodies (Origene, TA810067 and TA810055) against human L-SIGN, which is consistent with findings of previous reports (refs. 34, 35; Figure 4; and Supplemental Figure 3A). However, unlike prior reports (19, 36), L-SIGN was not detected in other blood vessels, such as the central vein, portal vein, hepatic artery in the liver, or high endothelial venules and arterioles in the lymph nodes. It was also not detected in airway epithelial cells and other cells in the lung or in LECs in other tissues, such as intestine, heart, and kidney (Supplemental Figure 3B). Of note, we did not detect ACE2 in LSECs and in lymph node LECs based on immunostaining with 2 different antibodies (Abcam, ab89111, and R&D Systems, AF933; Supplemental Figure 4A and ref. 37). Thus, our data indicate that L-SIGN but not ACE2 is uniquely expressed on LSECs and on LECs in the lymph node.

We used flow cytometry to measure the expression of endogenous L-SIGN and ACE2 in cultured primary or transformed endothelial cell lines. HEK293T cells transfected with either L-SIGN-flag or ACE2-myc3 were used as positive controls. Neither receptor was detected on immortalized human umbilical cord vein endothelial cells (HUVECs), a hybrid endothelial cell line EAhy926, or immortalized human dermal microvascular blood endothelial cells (DMECs; Supplemental Figure 4, B–D). A previous mRNA microarray study reported L-SIGN mRNA induction in human endothelial cells upon stimulation with TNF-α or IL-4 (38). However, we did not detect endogenous L-SIGN expression in human endothelial cell lines after TNF-α or IL-4 challenge (Supplemental Figure 4, B–D). Furthermore, these cells were not susceptible to CoV-2–type infection (Supplemental Figure 4E). These data are consistent with our tissue staining results, which indicate that L-SIGN is not expressed by most vascular endothelial cells.

We then examined cultured primary LSECs (passage 3) and LECs (passage 7). CD31, an endothelial cell marker, was expressed in these cells. However, endogenous L-SIGN was not detected (Supplemental Figure 4F). The loss of expression of endogenous L-SIGN on these primary cells is likely caused by the in vitro culture conditions as previously noted (39). Nevertheless, LSECs transduced with lentivirus to express L-SIGN-flag were susceptible to CoV-2–type infection (Supplemental Figure 4G).

### L-SIGN is a receptor for authentic SARS-CoV-2 virus.

**Figure 5:**
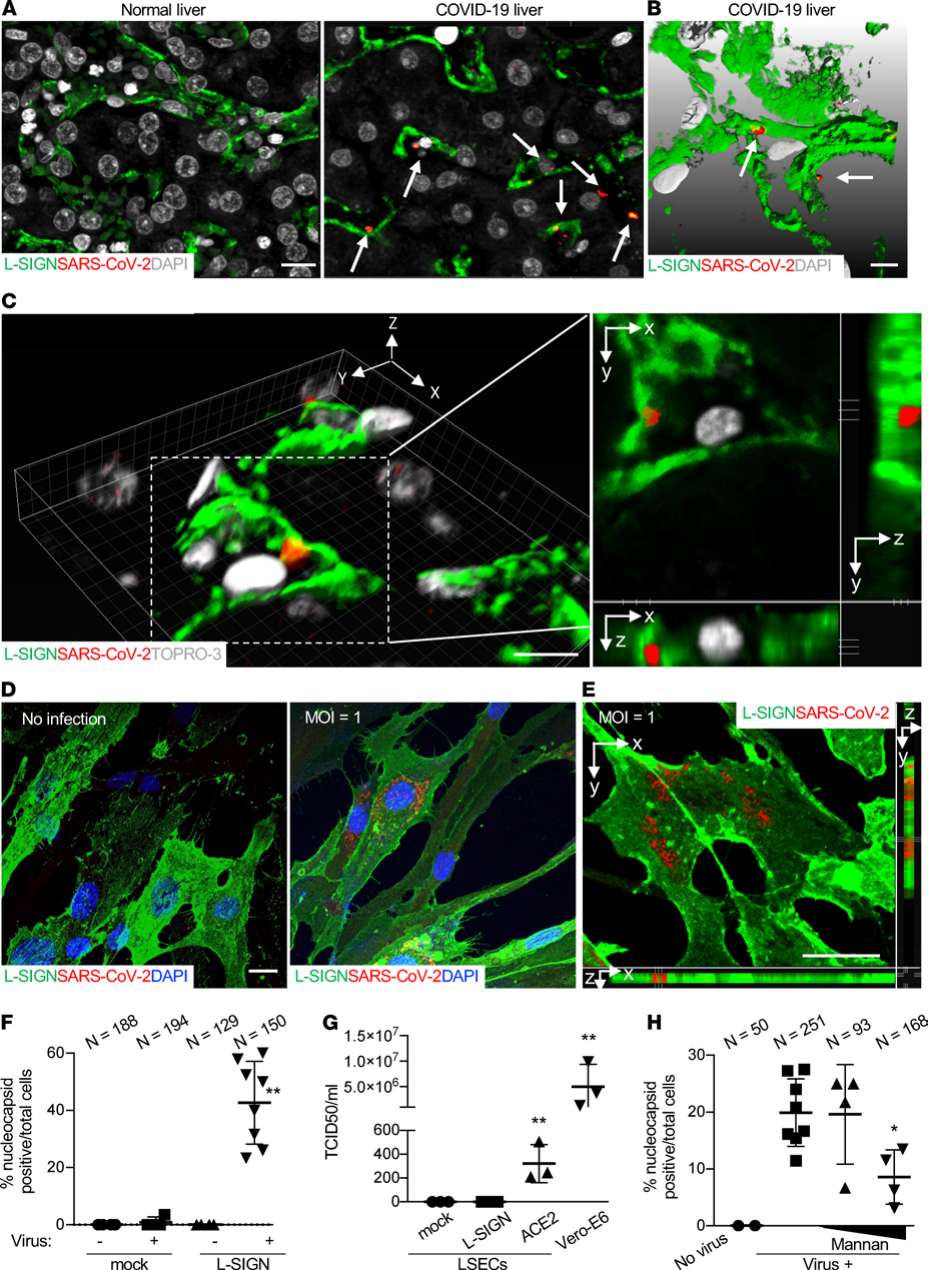
L-SIGN is a receptor for SARS-CoV-2 virus. (**A**) Representative confocal microscopy images of SARS-CoV-2 nucleocapsid protein in LSECs from a COVID-19 liver autopsy sample. An uninfected normal human liver autopsy sample was used as a negative control. LSECs were L-SIGN^+^ (green). Arrows mark SARS-CoV-2 (red). DAPI (gray), nuclear staining. Scale bar: 10 μm. (**B**) Representative of 3D rendering of *Z*-stacked microscopy of liver sections from COVID-19 liver autopsy samples. Scale bars: 3 μm. Arrows mark SARS-CoV-2 protein particles (red) inside LSECs. Scale bar: 5 μm. (**C**) Representative 3D reconstructed confocal microscopy images of sections from a COVID-19 liver autopsy sample (left). Images on the right show orthogonal *xy*, *xz*, and *yz* projections of the dotted area to reveal the virus staining inside the LSEC. Scale bars: 10 μm. The results represent data from biopsy of autopsy samples of 4 independent patients with COVID-19 and 3 uninfected patients. (**D**) Representative confocal microscopy images of cultured L-SIGN-flag–expressing LSECs infected with authentic SARS-CoV-2 (MOI = 1). Scale bar: 5 μm. Blue (DAPI), nuclear staining; green, L-SIGN; red, nucleocapsid protein. (**E**) A 3D microscopy view of L-SIGN-flag–expressing LSECs infected with authentic SARS-CoV-2 (MOI = 1). Scale bar: 5 μm. Green, L-SIGN; red, nucleocapsid protein. (**F**) Percentage of nucleocapsid protein^+^ cells per total cells in 4–8 ×40 fields in mock- or L-SIGN-flag–transduced LSECs. (**G**) Median tissue culture infectious dose (TCID50) virus titer assay of supernatants 2 days after infection in mock-, L-SIGN-flag–, or ACE2-myc3–transduced LSECs. Infected Vero-E6 cells were positive control in terms of their permissiveness for high viral productivity. (**H**) Percentage of nucleocapsid protein^+^ cells per total L-SIGN-flag–transduced LSECs in the presence of mannan (100 and 500 μg/ml) in 4–8 ×40 fields in each group. *P < 0.01; **P < 0.0001. Bars indicate the mean; error bars represent mean ± SD. All experiments were at least repeated 3 times.

SARS-CoV-2 can be detected in many tissues, including the lungs, pharynx, heart, brain, kidneys, lymphatic drainage, blood, and liver (40–43). To determine the clinical significance of the interaction between SARS-CoV-2 and L-SIGN, we first examined if SARS-CoV-2 was present in LSECs from formalin-fixed paraffin-embedded liver autopsy samples from patients with COVID-19. In comparison with uninfected human liver autopsy samples, we found increased expression of L-SIGN and detected SARS-CoV-2 with an anti-SARS-CoV-2 nucleocapsid antibody (Invitrogen, MA1-7404) inside patient LSECs (Figure 5, A–C).

To demonstrate that L-SIGN is a receptor for authentic SARS-CoV-2 virus, we infected L-SIGN-flag–expressing or –nonexpressing LSECs with authentic SARS-CoV-2 virus with multiplicity of infection (MOI) of 1 (MOI refers to the number of viruses that are added per cell during infection) for 18 hours and observed L-SIGN–dependent authentic SARS-CoV-2 infection in LSECs at 48 hours after infection (Figure 5, D–F). Based on 50% tissue culture infectious dose assay of supernatant, productive infection, which indicates viral amplification within infected cells, was elicited by authentic SARS-CoV-2 infection in ACE2-myc3–transduced LSECs; however, it was not elicited in either mock- or L-SIGN-flag–transduced LSECs (Figure 5G).

L-SIGN is known to capture certain viral particles and trans-infect adjacent cells (20, 44). To test whether L-SIGN promotes transmission of SARS-CoV-2 to blood cells, we incubated human CD45^+^ peripheral blood cells with L-SIGN-flag–transduced LSECs that were preinfected for 2 hours with pseudo-typed SARS-CoV-2. We detected CoV-2 infection in LSECs but not in CD45^+^ blood cells, indicating that L-SIGN does not mediate trans-infection in blood cells in this experimental setting (Supplemental Figure 5A).

We then asked if blocking L-SIGN function abolishes SARS-CoV-2 infection. To test this, we used a pseudo-CoV-2–type model. L-SIGN-flag–expressing HEK293T cells were treated with an antibody against human L-SIGN (anti–L-SIGN, R&D Systems, MAB162), with mannan to block mannose-dependent interactions or with L-SIGN/Fc recombinant protein (Supplemental Figure 5, B–E). As negative controls, we used isotype-matched control IgG or Fc control protein. Anti–L-SIGN, mannan, or L-SIGN/Fc recombinant protein dose-dependently blocked L-SIGN–mediated CoV-2–type infection but not control VSVG-type infection, indicating specific inhibition of L-SIGN–mediated CoV-2 infection. CoV-2–type infection was not blocked by negative controls (Supplemental Figure 5, B–E). These data demonstrate that infection requires interactions of a mannose glycan on the CoV-2–type spike protein with L-SIGN, suggesting blocking L-SIGN as a potential therapeutic option. Indeed, treatment of L-SIGN–transduced LSECs with mannan dose-dependently blocked authentic SARS-CoV-2 infection (Figure 5H).

### LSECs from liver autopsy samples from patients with COVID-19 exhibit signs of activation and increased procoagulant activities.

**Figure 6:**
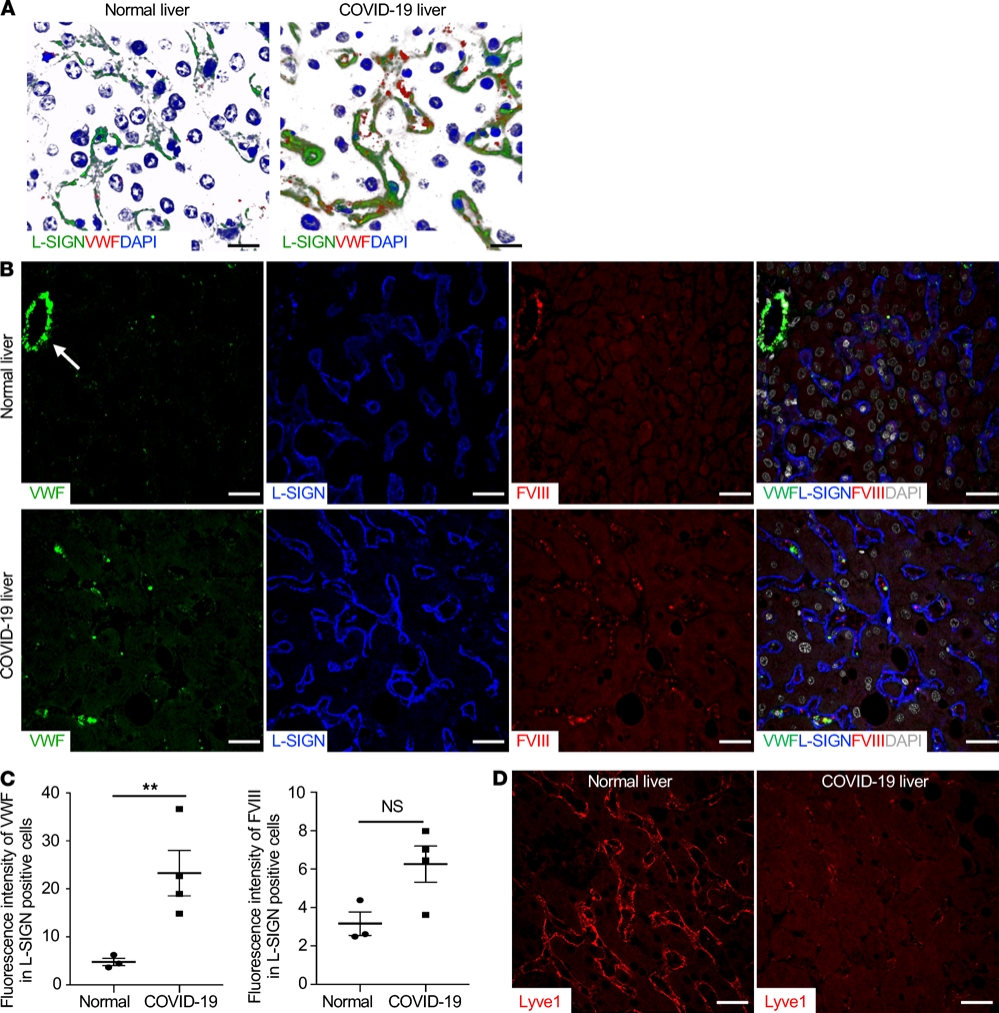
LSECs from liver autopsy samples from patients with COVID-19 express increased levels of L-SIGN, vWF, and FVIII. (**A**) Representative 3D rendering of *Z*-stacked confocal microscopy images of liver sections from normal human and COVID-19 liver autopsy samples. Scale bars: 20 μm. Green, L-SIGN; red, vWF; blue (DAPI), nuclear staining. (**B**) Representative single-color or merged-color confocal images of immune-stained human liver sections. Arrow indicates central vein. L-SIGN marks LSECs. vWF (green), L-SIGN (blue), FVIII (red), DAPI (white). Scale bar: 20 μm. (**C**) Quantification of relative mean fluorescence intensity of vWF or FVIII associated with L-SIGN^+^ LSECs. **P < 0.01. For all analyses, *n =* 4. Bars indicate the mean; error bars represent mean ± SEM. (**D**) Representative confocal microscopy images of Lyve1 (red) in a COVID-19–infected liver autopsy sample. An uninfected normal human liver autopsy sample was used as a positive control. Scale bar: 10 μm.

LSECs are major cells in the liver and are the primary source of the clotting factors vWF and FVIII. We detected a significant increase in expression of vWF in the patient LSECs relative to that in uninfected controls (Figure 6, A and C, and refs. 7, 21, 22). In addition, expression of FVIII in the patient LSECs relative to that of uninfected controls also showed an increased trend, even though it was not statistically significant (Figure 6, B and C). Coexpression of *CLEC4M* (encoding L-SIGN) with *vWF*, *F8*, and *PLAT* (encoding key coagulation factors vWF, FVIII, and t-PA, respectively) in LSECs was validated by analyzing published single-cell RNA-sequencing data from human liver (Supplemental Figure 6 and ref. 45). Interestingly, Lyve1, a marker for LSECs, was barely detectable in the patient LSECs relative to that in uninfected controls (Figure 6D). Reduced expression of Lyve-1 has been associated with activation of LSECs (46, 47).

## Discussion

Our data demonstrate that L-SIGN is a SARS-CoV-2 receptor that interacts with high-mannose–type N-glycans on the viral spike protein. L-SIGN was highly expressed on LSECs and on LECs in the lymph node of human biopsy or autopsy samples. We detected SARS-CoV-2 in LSECs from COVID-19 autopsy liver samples. Most importantly, our data demonstrate that L-SIGN mediates infection of authentic SARS-CoV-2 into endothelial cells, which was correlated with their procoagulant activities.

L-SIGN is a type II transmembrane protein. The phylogenetic tree suggests that gene duplication of DC-SIGN in the Old World monkey has given rise to L-SIGN (33). L-SIGN is a C-type lectin receptor that mediates infections of Ebola virus, Marburg virus, Japanese encephalitis virus, HIV-1, and hepatitis C virus via interactions with viral envelope glycoproteins. SARS-CoV-2 is related to SARS-CoV, which caused the first coronavirus epidemic in 2003 (48). SARS-CoV-2 and SARS-CoV have similar spike proteins. However, our data showed the differences in important N-glycosylation sites in these spike proteins recognized by L-SIGN (Supplemental Figure 2G), consistent with some published studies (48, 49). Indeed, mutagenesis of N330 in the RBD of SARS-CoV, equivalent to Asn343 of SARS-CoV-2, does not affect L-SIGN–mediated SARS-CoV infection (49). Unlike the case of SARS-CoV, our data demonstrated that L-SIGN binds to a high-mannose–type N-glycan, at least on the RBD of the spike protein to mediate interaction of SARS-CoV-2 with host cells. The molecular basis of the difference in the L-SIGN recognition domain of spike protein between SARS-CoV and SARS-CoV-2 remains to be studied (48). In addition, proteolytic activation of the spike protein by TMPRSS2 and lysosomal proteases are important for SARS-CoV-2 entry mediated by ACE2. It remains to be determined if proteolytic activation of the spike protein is required for L-SIGN–mediated SARS-CoV-2 infection.

Studies of N-glycans on RBD Asn343 yielded discrepant results, probably due to different expression systems used to produce recombinant spike protein for glycan profiling (10, 11). However, a few papers have shown that high-mannose–type N-glycans on Asn343 are predominant in the entire spike protein and partial domain of spike protein (10, 14). Our glycan structural analysis and Endo-H assay indicate that high-mannose–type N-glycans are common on the spike protein. Recently, N-glycan on Asn343 in spike protein has been reported to be critical for open-up conformational change so that RBD can be exposed to ACE2 (17). We have shown, as has a previous report, that mutagenesis of Asn343 to Gln in spike protein drastically reduced ACE2-mediated infection (Figure 3F and ref. 26). Our functional assays show that the high-mannose–type N-glycan on Asn343 on spike/Fc, which only comprises RBD of SARS-CoV-2 spike protein, is critical for binding to L-SIGN but not to ACE2 (Figure 2E). These differential requirements of Asn343 in binding to ACE2 between the entire spike protein and spike/Fc are presumably because spike/Fc does not need conformational change for binding to ACE2.

ACE2 was the first documented entry receptor for SARS-CoV-2 (50, 51). In human lungs, ACE2 is expressed on alveolar epithelial type II cells, which mediate virus entry and replication that causes acute alveolar epithelial cell injury (4). Other than the lung, ACE2 is also found in the kidney, small intestine, and heart, which possibly contributes to pathologies in these organs. Coagulopathy and thrombosis are common complications of patients with severe COVID-19 (5, 7). Although the causes of coagulopathy in severe COVID-19 are almost certainly multifaceted, including inflammatory responses (52, 53), previous studies have demonstrated a role for endothelial cell injury or dysfunction (54). However, there is no convincing evidence that ACE2 is expressed in vascular endothelial cells (54, 55). In our study, ACE2 was not detected in vascular endothelial cells in different human tissues or in multiple human endothelial cell lines from different origins.

Vero-E6 cells are defective in interferon responses, and, therefore, they are commonly used to amplify virus titer. However, LSECs would have an intact interferon response. Indeed, viral titer from ACE2-myc3–transduced LSECs was much lower than that from Vero-E6 cells, although the infections were performed differently (MOI = 0.01, 18 hours; Figure 5G). This is probably because authentic SARS-CoV-2 infection in LSECs might cause interferon responses following cytotoxicity that crippled the ability to release a large amount of virus.

SARS-CoV-2 viral genome was detected in COVID-19 liver (40, 56). Recent large postmortem liver biopsy studies showed liver sinusoidal dilation (57, 58) and frequent microthrombi associated with sinusoid (56). A recent ultrastructural analysis of liver biopsies from patients with COVID-19 also show virions within LSECs (59). Consistent with our in vitro data that shows no replication in infected LSECs (Figure 5G), failure of in situ hybridization using SARS-CoV-2 “antisense strand” to detect viral “replication” indicates no viral replication in COVID-19 livers (59). These results, together with our immunostaining data (Figure 5), support that LSECs are a target of SARS-CoV-2 with nonproductive infection.

Markers of endothelial cell activation, such as increased circulating vWF and FVIII, have been observed in patients with severe COVID-19 in hospital intensive care units; these markers are correlated with mortality (7). During severe inflammation, many factors contribute to endothelial cell activation. Our data suggest that interactions of SARS-CoV-2 with L-SIGN on LSECs can mediate endothelial injury. Notably, we detected considerably higher levels of L-SIGN on LSECs in livers from patients with COVID-19 than in those from uninfected controls. Given that our pseudo-typed viral infection assay indicated positive correlation of L-SIGN expression level and infection (Figure 1F), it is plausible to speculate that LSECs with elevated L-SIGN expression in patients with COVID-19 are infected preferentially. The LSECs from patients with COVID-19 lost expression of Lyve-1, a sign of liver inflammation, but exhibited procoagulant activities, suggesting endothelial dysfunction (Figure 6 and refs. 7, 46). LSECs, together with lymph node LECs, are primary sources of FVIII (21, 23), which pairs with vWF as an essential procoagulant (21). Coagulation changes in patients with the most severe COVID-19 are different from typical sepsis-related disseminated intravascular coagulopathy because of less prominent thrombocytopenia and consumptive coagulopathy, suggestive of an unappreciated mechanism (7). In addition, hyperfibrinolysis (increased D-dimer levels) was prominent in patients with COVID-19. Other than *vWF* and *FVIII*, *t-PA* is also highly expressed in LSECs (Supplemental Figure 6). Activated endothelial cells release t-PA (60), which could cause the burst of plasmin and subsequent increased D-dimer in patients with COVID-19. Enlarged livers have been noted in patients with COVID-19 (61). LSECs comprise a significant portion of nonparenchymal cells in the liver, the largest solid organ in humans (62). Our results suggest that L-SIGN–mediated activation of vWF- and FVIII-enriched LSECs may contribute to the pathogenesis of coagulopathy and thrombosis in patients with severe COVID-19 (7).

Multiple animal models of SARS-CoV-2 infection have been reported, ranging from rodents to Old World and New World monkeys (50, 51, 63–65). These animal models display consistent pulmonary inflammatory changes, but none exhibits obvious signs of coagulopathy or thrombosis. This may be explained by the fact that L-SIGN is not expressed in these nonhuman primates. Other experimental models are needed to identify additional unappreciated factors contributing to COVID-19–related coagulopathy in humans. Based on our results, it is plausible that SARS-CoV-2 infection of LSECs and LECs through L-SIGN leads to endothelial cell activation and secretion of vWF and FVIII into circulation, which may synergize with ACE2-mediated infection to cause coagulopathy in humans (21, 33, 66). Indeed, ligand-engagement of L-SIGN is known to trigger mitogen-activated protein kinase cascades (67, 68). Our data also indicate that caution needs to be taken in extrapolating results from animal models of SARS-CoV-2 infection to the human disease.

Relative to ACE2-mediated infection, we found that L-SIGN–mediated authentic SARS-CoV-2 infection in LSECs does not significantly produce new virus. Previous studies show that SARS-CoV infection in antigen-presenting cells, such as macrophages or dendritic cells, is not productive but efficiently primes T lymphocytes (69). This suggests that the intracellular route of entry mechanisms between ACE2 and L-SIGN–mediated infection of SARS-CoV-2 differs. ACE2-mediated infection causes productive infection by replicating viral genome in the cytosol and assemblies of viral progenies in the secretory pathway, whereas viral particles entered host cells mediated by L-SIGN are possibly transported to the endo/lysosome, resulting in destruction of viral particles and stimulation of MHC-II–dependent antigen presentation to develop acquired immunity. Indeed, LSECs have an antigen-presentation function in addition to production of coagulation factors (70). Therefore, L-SIGN–mediated infection in LSECs may lead to augmented procoagulant activity and modulation of immune responses.

During preparation of this manuscript, two manuscripts relevant to L-SIGN as a new receptor for SARS-CoV-2 appeared in *bioRxiv*. One manuscript reported that L-SIGN is expressed on blood endothelial cells in the lung (36). However, we could not detect L-SIGN expression in blood endothelium of many types of human tissues, including the lung, despite utilizing two different immunostaining methods, multiple approaches for antigen retrieval, and 2 independent anti-human L-SIGN antibodies that recognize different epitopes. In addition, we did not detect CoV-2–type infection in immortalized HUVECs and DMECs (Supplemental Figure 4). Therefore, our results do not support L-SIGN as a receptor for SARS-CoV-2 on most blood endothelial cells. The other manuscript reported that L-SIGN is 1 of 4 C-type lectin receptors on innate immune cells that can bind to SARS-CoV-2 (71). Neither of these studies examined expression of L-SIGN in LSECs or lymph node LECs in human tissues or considered its role in COVID-19–related coagulopathy. Nevertheless, results in these preprints demonstrated direct interaction between purified recombinant spike protein and L-SIGN using in vitro binding assays (36, 71).

A study that recently appeared in *bioRxiv* reports that L-SIGN was scored as one of interactors of SARS-CoV-2 spike protein using a genomic receptor screening method (72). Although this study does not provide any functional analysis, its unbiased screening approach strengthens our finding that L-SIGN is a receptor for SARS-CoV-2.

Recently, heparan sulfate proteoglycan (HSPG) and neuropilin-1 (NRP-1) were reported to facilitate SARS-CoV-2 infection (25, 73, 74). NRP-1 is thought to potentiate ACE2-mediated SARS-CoV-2 infection in respiratory and olfactory epithelium by promoting the interaction of the spike protein with ACE2 (25, 74). Interestingly, *EXT1*, an enzyme responsible for synthesizing HSPG, is scarcely detected in LSECs, whereas *NRP-1* is uniquely detected in LSECs in human livers (Supplemental Figure 6). Given that ACE2 is not expressed in LSECs, NRP-1 may also facilitate L-SIGN–mediated SARS-CoV-2 infection.

In summary, our data not only reveal L-SIGN expressed by transfection or lentiviral transduction as a receptor for SARS-CoV-2, but also show its potential biological role in the pathogenesis of coagulopathy in patients with severe COVID-19. In addition, we found that interactions between SARS-CoV-2 and L-SIGN could be blocked by anti–L-SIGN antibody, by mannan, or by recombinant L-SIGN/Fc protein. These results suggest potential therapeutic options to treat severe COVID-19 infection.

## Methods

Further details can be found in the Supplemental Methods.

### CoV-2–type virus production, infection inhibition assay, and trans-infection assay.

HEK293T cells (1 × 10^6^ cells) were plated on 6-well plates. On the following day, pHIV-ZsGreen (3.3 μg), psPAX2 (2.5 μg), and SARS-CoV-2-spike (1 μg) were cotransfected, and after 15 hours, media were replaced with fresh media. On the following day, culture media containing virus were aseptically centrifuged at 15,000*g* for 10 minutes at room temperature, and after adding polybrene (2 μg/ml), CoV-2–type virus–containing medium was added to target cells. After 48 hours, infection efficiency, defined by ZsGreen expression, was determined by flow cytometry. VSVG-type pseudo-typed virus was generated by cotransfecting of pHIV-ZsGreen (3.3 μg), psPAX2 (2.5 μg), and pMD2.G (1.67 μg) as positive control. For the infection inhibition assay, mouse anti-human L-SIGN mAb (10 and 2 μg/ml) or mannan (500 and 100 μg/ml) were added at the same time as the infection. The L-SIGN/Fc recombinant protein (50 and 10 μg/ml, corresponding to 1 and 0.2 nmol/ml) were preincubated with the virus-containing media for 15 minutes at room temperature before initiating the infection. Isotype-matched control IgG (10 and 2 μg/ml) and Fc control (1 and 0.2 nmol/ml) were used as negative controls. For trans-infection assay, L-SIGN-flag–transduced LSECs were preincubated with SARS-CoV-2 pseudo-typed CoV-2 for 2 hours at 37°C. After washing twice, LSECs were cocultured with the same number of human peripheral blood cells. At 48 hours after coculture, detached cells were stained with APC-anti-human CD45 mAb, and ZsGreen expression was analyzed in CD45^+^ (blood cells) and CD45^–^ (LSECs) populations.

### SARS-CoV-2 procurement and handling.

SARS-CoV-2 isolate USA-WA1/2020, which belongs to the ancestral Wuhan strain, was deposited by the Centers for Disease Control and Prevention and obtained through BEI Resources Repository, National Institute of Allergy and Infectious Diseases, NIH (NR-52281). Cellular studies with SARS-CoV-2 isolate USA-WA1/2020 were conducted in the University of Oklahoma Health Sciences Center High Containment Biosafety Level 3 Laboratory Core under Institutional Biosafety Committee–approved biosafety protocol 100492. All viral infections were performed under BSL-3 conditions at negative pressure by personnel fitted with personal protective gear that include Tyvek suits connected with personal powered air-purifying respirators. Experimental samples were only removed from the BSL-3 facility after SARS-CoV-2 was deactivated by established approaches that include heat deactivation (30 min at 65°C) or fixation (4 % paraformaldehyde for 20 min).

### Authentic SARS-CoV-2 virus infection.

LSECs with or without expression of L-SIGN were plated on glass coverslips in 24-well plates (1 × 10^5^ cells/well). On the following day, authentic SARS-CoV-2 virus was added to each well at MOI of 0.1 or 1. After 18 hours, media were replaced with fresh media. For the infection inhibition assay, mannan (500 and 100 μg/ml) were added at the same time as the infection. Forty-four hours after infection, culture media containing virus were aseptically collected and inactivated by heating (65°C, 30 min). Cells were fixed with 4% PFA for 20 minutes at room temperature for immunofluorescence (75).

### Flow cytometric analysis.

To examine spike/Fc binding to cells, plasmids coding for human ACE2, L-SIGN, and DC-SIGN were transfected in HEK293T cells, and after 48 hours, cells were incubated with spike/Fc (50 μg/ml) in HBSS (Corning, 21-023-CV) on ice for 1 hour. After washing with HBSS, cells were further stained with Alexa Fluor 488–conjugated anti-human IgG (Jackson ImmunoResearch, 709-545-098) on ice for 30 minutes. After washing again, cells were analyzed on FACSCelesta (BD), and data were analyzed using FlowJo software. To confirm specificity of spike/Fc to L-SIGN–expressing cells, cells were pretreated with anti–L-SIGN mAb (10 μg/ml) for 10 minutes and then stained with spike/Fc. To examine L-SIGN/Fc binding to cells, the plasmid coding for spike was transfected in HEK293T cells, and after 48 hours, cells were incubated with L-SIGN/Fc (1 g/ml) on ice for 1 hour. Control Fc was used as a negative control. For the inhibition assay, L-SIGN/Fc was preincubated with mannan (1 μg/ml) for 15 minutes at room temperature. L-SIGN expression was examined using anti–L-SIGN–specific mAb (R&D Systems, MAB162). For lectin staining, cells were incubated with GNL (2 μg/ml) on ice for 1 hour. After washing with HBSS, cells were further stained with PE-conjugated streptavidin (Jackson ImmunoResearch, 016-110-084) on ice for 30 minutes. Double staining of CD31 and L-SIGN was done by staining cells with rabbit anti-human CD31 pAb (Santa Cruz Biotechnology, sc-8303) and anti–L-SIGN mAb followed by secondary staining with fluorescence-conjugated antibodies.

### Statistics.

Statistical tests were performed using Prism software (GraphPad). Two-sided, two-tailed Student’s *t* tests were performed to assess the statistical significance of differences between 2 groups after the data were confirmed to fulfill the criteria of normal distribution and equal variance. One-way ANOVA was used to analyze the significance of differences among 3 or more groups. Differences were considered statistically significant at *P* < 0.05. No data points were excluded from the analysis performed in this study.

### Study approval.

Uses of deidentified formalin-fixed paraffin-embedded biopsy or autopsy patient tissues in this study was reviewed and approved by the Institutional Review Board of the Oklahoma Medical Research Foundation and does not qualify as human subject research.

## Author contributions

YK and LX conceived and designed the experiments, interpreted data, and wrote the manuscript. YK, JLL, LG, JMM, HS, BS, CMH, SAAH, PA, and JFP performed experiments and analyzed data. ZY, AB, JCL, JA, YCH, RSS, RSM, ARR, and FL provided deidentified human tissue samples, cell lines, key reagents, or comments. RPM commented on the project and contributed to the manuscript preparation.

## Supplementary Material

Supplemental data

## Version 1 07/22/2021

Electronic publication
