# Supplementary material for: L-SIGN is a receptor on liver sinusoidal endothelial cells for SARS-CoV-2 virus
Source: JCI Insight. 2021 Jul 22;6(14):e148999. doi: 10.1172/jci.insight.148999 (PMC8410055; doi:10.1172/jci.insight.148999)
Supplement: Supplemental data [file jciinsight-6-148999-s200.pdf]

**Supplemental Data**

**L-SIGN is a receptor on liver sinusoidal endothelial cells for SARS-CoV-2 virus**

Yuji Kondo,[1] Jason L. Larabee,[2] Liang Gao,[1] Huiping Shi,[1] Bojing Shao,[1] Christopher Hoover,[1,3] J. Michael McDaniel,[1] Yen Chun Ho,[1] Robert Silasi-Mansat,[1] Stephanie A. Archer-Hartmann,[4] Parastoo Azadi,[4] R. Sathish Srinivasan,[1] Alireza R. Rezaie,[1,3] Alain Borczuk,[5] Jeffrey C. Laurence,[6] Florea Lupu,[1,7] Jasimuddin Ahamed,[1,8] Rodger P. McEver,[1,3] James F. Papin,[7] Zhongxin Yu,[7] and Lijun Xia[1,3]

[1]Cardiovascular Biology Research Program, Oklahoma Medical Research Foundation, Oklahoma City, OK 73104, USA. [2]Department of Microbiology and Immunology, [3]Department of Biochemistry and Molecular Biology, University of Oklahoma Health Sciences Center, Oklahoma City, OK 73104, USA. [4]Complex Carbohydrate Research Center, University of Georgia, Athens, GA 30602, USA. [5]Department of Pathology and Laboratory Medicine, [6]Division of Hematology and Medical Oncology, Weill Cornell Medicine, New York, NY 100065, USA. [7]Department of Pathology, [8]Department of Physiology, University of Oklahoma Health Sciences Center, Oklahoma City, OK 73104, USA

Address correspondence to:

Lijun Xia, M.D., Ph.D., Cardiovascular Biology Research Program, Oklahoma Medical Research Foundation, 825 N.E. 13th Street, Oklahoma City, OK 73104; Phone: 405-271-7892. Fax: 405-271-3137. Email: lijun-xia@omrf.org

**Supplemental methods**

*Cell culture*. COS-7 cells and HEK293T cells purchased from ATCC (Cat# CRL-1651, CRL-3216) were grown in DMEM (Corning # 10-013-CV) supplemented with 10% FBS, 100 U/ml penicillin, and 100 μg/ml streptomycin as previously described (1). Human dermal microvascular endothelial cells and human liver-derived sinusoid endothelial cells purchased from Lonza (Cat# CC-2543, HLECP2) were grown in EGM-2 medium (Lonza Cat# CC-3162).

*Reagents*. Anti-human L-SIGN mouse monoclonal antibody (mAb) for western blotting, flow cytometry, and infection inhibition assay, and anti-human ACE2 antibody for flow cytometry were purchased from R&D Systems (Cat# MAB162, AF933). Anti-human ACE2 mouse mAb for tissue immunohistostaining was purchased from Abcam (Cat# ab89111). Anti-human L-SIGN Abs for tissue immunohistostaining were purchased from ORIGENE (Cat# TA810067, TA810055 and Sigma Cat# AV42396). Anti-flag and anti-myc antibodies were obtained from Santa Cruz Biotechnology (Cat# sc-166355, sc-40). Anti-coagulation factor VIII antibody was purchased from LSBio (Cat# LS-B2979). Anti-SARS nucleocapsid protein antibodies were from Novus Biologicals (Cat# NB100-56576). PNGaseF, Endo-H and α1-2,3,6 mannosidase were purchased from NEB (Cat# P0704S, P0702S, P0768S). Neuraminidase was from Millipore Sigma (Cat# 10269611001). Mannan was purchased from Sigma (Cat# M7504). Kifunensine was purchased from Cayman Chemical (Cat# 10009437). Anti-human CD45 mouse mAb for flow cytometry was purchased from Biolegend (Cat# 304011).

*Plasmids*. Plasmids of human ACE2, SARS-CoV2-Spike, pLVX-M-puro were purchased from Addgene (Cat# 1786, 145032, 125839). The full cDNA sequence of the human ACE2 was subcloned into pcDNA3.1(+)/myc3 to express C-terminal myc3-tagged human ACE2 as previously described (1). Plasmids of C-terminal flag-tagged L-SIGN, DC-SIGN, and TMPRSS2

were purchased from Genscript (Cat# OHu19035, OHu23087, OHu13675). The cDNA sequence of the extracellular domain of human L-SIGN (residues 78–399) was sub-cloned into pFUSEN-hG2Fc plasmid (InvivoGen Cat# pfcn-hg2) to express N-terminal human Fc-tagged human L-SIGN (L-SIGN/Fc) recombinant soluble protein. The cDNA sequence of the SARS-CoV2 Spike RBD (residues 333–529) was sub-cloned from pcDNA3-SARS-CoV-2-S-RBD-Fc (Addgene Cat# 141183) into pFUSE-hIgG2-Fc2 plasmid (InvivoGen Cat# pfuse-hfc2) to express C-terminal human Fc-tagged Spike RBD (Spike/Fc) recombinant soluble protein. The cDNA sequence of the L-SIGN was sub-cloned into pLVX-M-puro. N343Q-carrying Spike/Fc mutant expression vectors, mutated EPN motif-carrying L-SIGN expression vectors, and mutated endocytosis motif-carrying L-SIGN expression vectors were generated by inverse PCR using KOD Xtreme DNA polymerase (Millipore Sigma Cat# 71975). pHIV-ZsGreen plasmid was a kind gift from Dr. Bryan E. Welm (University of Utah). Plasmids of psPAX2 and pMD2.G were purchased from Addgene (Cat# 12260, 12259).

*Viral Stocks*. SARS-CoV-2 was grown for up to 3 passages in Vero-E6 cells (ATCC: CRL-1586) cultured in DMEM (Dulbecco's Modified Eagle Medium) containing 5% FBS and antibiotics (0.1 units penicillin and 0.1 mg/mL streptomycin) and maintained at 37°C with 5% CO2. To passage SARS-CoV-2, Vero-E6 cells were grown in a T-150 flask to 50 % confluence (~10 million cells) and inoculated with SARS-CoV-2 at a MOI of 0.001. This infection was carried out in 3 ml of DMEM (no FBS) for 1 h at 37°C with gentle mixing every 10 min. Following this incubation, DMEM (5% FBS and antibiotics) was added bringing the volume to 30 ml. The culture was continued for 48 h and then virus was harvested by collecting cell culture supernatants, centrifuging and storing aliquots at -80°C. All experiments involving viral infection were conducted using virus from the same viral stock. SARS-CoV-2 was subsequently

titrated using the 50% tissue culture infectious dose (TCID50) method. With the TCID50 method, VERO E6 cells were seeded at 10,000 cells per well in a 96-well plate immediately before the assay. SARS-CoV-2 was then serially diluted starting with a 1:10 dilution across the plate. After 96 h, each well was visually inspected under a microscope to determine which wells were positive for SARS-CoV-2 induced cytopathic effects (CPE). At each dilution, the number of virus positive wells was recorded and these results were used to calculate TCID50/ml using the Reed and Muench Method. The TCID50 method was also used to detect and quantify productive infections in endothelial cells infected with SARS-CoV-2.

*Protein purification*. COS-7 cells in 20 x 100 mm dishes transfected with plasmid by PEI-max (MW 4,000) (Polysciences, Inc. Cat# 24885) were cultured for 3 days in serum-free Opti-MEM1 media (Invitrogen Cat# 51985034) containing ITS-X (Gibco Cat# 51500056), glutamine/penicillin/streptomycin, non-essential amino acids, sodium pyruvate, and 0.5% yeast extract (BD Biosciences Cat# 212750) as previously described (2). Culture medium was centrifuged at 3,000 g for 10 min, and supernatant was filtered through a 0.22 μm membrane. After adjusting pH with Tris-HCl buffer to pH 7.6, secreted L-SIGN/Fc, Spike/Fc, and Fc control were purified from the supernatant using Protein A agarose (Goldbio Cat# P-400-5). Afterward, the buffer was exchanged to PBS using a 30 K cut-off amicon filter tube (Millipore Corp Cat# UFC803008), protein concentration was measured by DC protein assay (Bio-Rad Cat# 5000111). Purity of proteins was examined by staining gel and membrane with Coomassie Brilliant Blue and Ponceau S solution, respectively. Silver staining was carried out according to manufacturer's protocol (Thermo Fisher Cat# 24612).

*Western blotting and lectin blotting*. Protein extracts from cultured cells or tissues were prepared using a 1% Triton X-100-based cell lysis buffer (Cell Signaling Technology Cat#

9803). Lysates were applied to an 8-12 % SDS-PAGE gel and separated proteins were transferred onto a PVDF membrane (Thermo Fisher Cat# 88518) as previously described (1). After blots were blocked with skim milk in TBS-0.05% Tween20 (TBS-T), the membranes were incubated with primary and HRP-conjugated secondary antibodies and developed with ECL systems (Thermo Fisher Cat# 34577). For the GNL lectin staining, transferred PVDF membranes were blocked with 5% BSA in TBS-T overnight at 4˚C, and incubated with biotin-conjugated GNL (1 μg/ml) in 5% BSA in TBS-T for 1 h at room temperature. After washing, the membrane was incubated with HRP-conjugated streptavidin (Jackson ImmunoResearch Cat# 016-030-084) in 5% BSA in TBS-T for 30 min at room temperature. Exposed X-ray films (MIDSCI Cat# BX57) were scanned and analyzed with ImageJ software (NIH).

*Histology and microscopy*. De-identified formalin-fixed paraffin-embedded biopsy or autopsy tissues from three uninfected patients as well as de-identified formalin-fixed paraffin-embedded autopsy samples of three COVID-19 patients were used.

For histology, paraffin sections (5 μm) were stained with hematoxylin and eosin as previously described (3). For immunohistochemistry, paraffin sections were deparaffinized and rehydrated. For antigen retrieval, heat-induced antigen retrieval was performed in 1 mM EDTA in 10 mM Tris buffer (pH 8.5) at 120°C in an autoclave for 3 min as previously described (4). In some experiments, paraffin sections were deparaffinized and incubated with 6 M guanidine hydrochloride for 30 min at room temperature. After wash, sections were then treated with 0.2 mg/ml trypsin for 30 min at 37°C. Endogenous peroxidase was inactivated by incubation with 3% hydrogen peroxide for 15 min at room temperature. For staining, sections were incubated with mouse anti-human L-SIGN mAb (Origene Cat# TA810067, TA810055) or rabbit anti-human L-SIGN polyclonal Ab (Sigma Cat# AV42396, 1:100) for 1.5 h at room temperature.

Secondary antibodies (5 μg/ml, HRP-conjugated goat anti-mouse IgG or HRP-conjugated goat anti-rabbit IgG) were added for 15 min at room temperature. The staining was visualized using diaminobenzidine substrate (Vector Laboratories Cat# SK-4100).

For immunofluorescent staining, paraffin sections were deparaffinized and rehydrated. Heat-induced antigen retrieval was performed by citrate buffer (pH 6.0) at 100°C for 20 min or by 1 mM EDTA in 10 mM Tris buffer (pH 8.5) at 120°C for 3 min. Paraffin sections were blocked for 3 h at room temperature and incubated with primary antibodies overnight at 4 °C. Secondary antibodies were added for 45 min at room temperature. Primary antibodies were to CD31 (Dako Cat# M0823, 1:20), VWF (Dako Cat# A0082, 1:200), SARS nucleocapsid protein (Novus Biologicals Cat# NB100-56576, 1:50), Lyve1 (Abcam Cat# ab219556, 1:3000), ACE2 (Abcam Cat# ab89111, R&D Systems Cat# AF933, 1:200), L-SIGN (Origene Cat# TA810067 and TA810055, 1:150), Factor VIII (LSBio Cat# LS-B2979, 1:200). Secondary antibodies were to Cy3-conjugated donkey anti-sheep IgG (Jackson ImmunoResearch Cat# 713-165-003), Cy3-conjugated donkey anti-mouse IgG (Jackson ImmunoResearch Cat# 715-165-150), Alexa Fluor 488-conjugated goat anti-mouse IgG (Jackson ImmunoResearch Cat# 115-546-062), Alexa Fluor 647-conjugated donkey anti-mouse IgG (Abcam Cat# ab150107), Alexa Fluor 488-conjugated goat anti-rabbit IgG (Jackson ImmunoResearch Cat# 111-546-003), Alexa Fluor 555-conjugated donkey anti-rabbit IgG (Abcam Cat# ab150074). The secondary antibodies were used at a concentration of 8 μg/ml.

For immunofluorescent staining, SARS-CoV-2 virus infected cells were fixed with 4% PFA for 20 min at room temperature followed by 3 times washes with PBS for each 5 min as previously described (1). After blocking with 3% BSA 3% donkey serum 0.05% Triton X-100 in PBS for 1 hr at room temperature, cells were incubated with goat anti-flag (2.5 μg/ml, Abcam

Cat# ab1238) and mouse anti-nucleocapsid protein antibodies (5 μg/ml, Invitrogen Cat# MA1-7404) for 2 hrs at room temperature. After 4 times washes with PBS for each 5 min, cells were incubated with Alexa Fluor488-conjugated donkey anti-goat IgG (Jackson ImmunoResearch Cat# 705-546-147) and Cy3-conjugated anti-mouse IgG (Jackson ImmunoResearch Cat# 715-165-150) for 1 hr at room temperature. After 4 times washes with PBS for each 5 min, cells were incubated with DAPI for 5 min, and mounted with mounting medium (Sigma, Cat# F4680).

A confocal microscope (Zeiss 710 Microscope System) was used for imaging based on our published methods (3, 4). Z-stack images were collected at 2-μm steps with sequential laser excitation to eliminate bleed-through and with confocal parameters selected to minimize the thickness of the calculated optical section using the Zeiss software ZEN. Volume images from the confocal image datasets were further processed with IMARIS software (Bitplane AG), and some images were presented as maximum intensity projections of the z-stacks. Mean fluorescent intensity measurements of VWF and FVIII were determined from independent 16-bit tiff images. The co-stained L-SIGN was used to outline the liver sinusoidal endothelial cells with the tracer tool in ImageJ. At least 4 40 x images per patient were analyzed for VWF or FVIII fluorescent intensity and the values were averaged. Tukey's test was used when comparing between groups.

*Glycan structural analysis*. Spike/Fc protein (0.15 mg) in PBS was sequentially mixed with 4 volumes of methanol, 1 volume of chloroform, and 3 volumes of water. After spinning at 15,000 g for 1 min, supernatant was discarded, and 4 volumes of methanol was added. After spinning at 15,000 g for 2 min, all solvent was discarded and samples were dried and dissolved in Ambic buffer. The solution was incubated with porcine trypsin (1:50) at 37˚C overnight and then heated at 100˚C for 5 min as previously described (5, 6). The peptides and glycopeptides were isolated by passing through a C18 spin column, and eluted by addition of 80% acetonitrile, before drying

by a SpeedVac. The dried glycopeptide eluate was treated with PNGase F to release the N-linked glycans by digestion overnight at 37˚C. The digested sample was then passed through a C18 sep pak cartridge to recover the *N*-glycans in the 5% acetic acid flow through. This fraction was then dried by lyophilization. The dried *N*-glycans were then permethylated for structural characterization by mass spectrometry. Briefly, the dried eluate was dissolved in dimethyl sulfoxide and methylated with NaOH and methyl iodide. The reaction was quenched with water and per-O-methylated carbohydrates were extracted with methylene chloride and dried under N2. The permethylated samples were dissolved in methanol and crystallized with α-dihydroxybenzoic acid (DHBA) matrix (20 mg/mL in 50% methanol/water). One μl of each sample and the matrix were combined on the plate and allowed to dry. Analysis was performed by MALDI-TOF-MS using AB SCIEX TOF/TOF 5800 (Applied Biosystem MDS Analytical Technologies). Data were collected from a mass ranging from 800 to 4,500 daltons. The focus mass was set for 2,500 for *N*-glycans. Following acquisition, the baseline was smoothed and the detection limits for peaks were set to 3 S/N. Purified recombinant Fc fusion proteins or culture supernatant from COS-7 and HEK293T cells transfected with plasmid encoding Spike/Fc were digested with PNGaseF and Endo-H as manufacturer's protocols, and analyzed by CBB staining, silver staining, western blotting or lectin blotting.

## Supplemental Figures

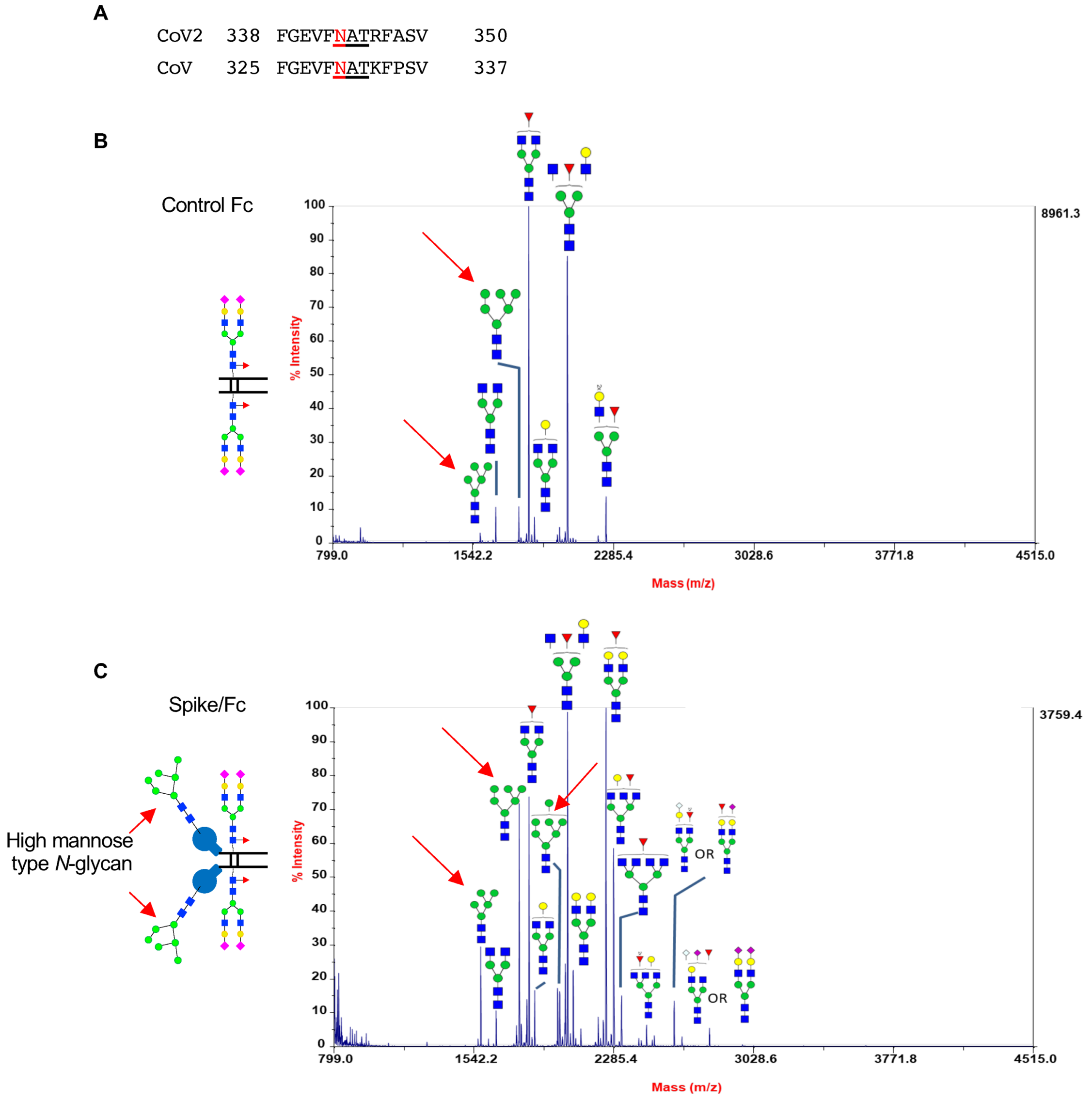


**Supplemental Figure 1. Spike/Fc has high-mannose type *N*-glycans.** (**A**) Primary sequence of receptor binding domain surrounding an *N*-glycosylation site (N343) of SARS-CoV-2 spike protein, which is conserved in receptor binding domain of SARS-CoV as marked with red. Underline is consensus *N*-glycosylation site (Asn - X - Ser/Thr). X cannot be Pro. (**B** and **C**) Glycan structure analyses of COS-7 cell-derived control Fc and Spike/Fc by MALDI-TOF mass spectrometry. The proposed structures of the glycans are shown with their corresponding ion peak in the MALDI-TOF MS spectrum. Arrows indicate major high-mannose type *N*-glycan structures.

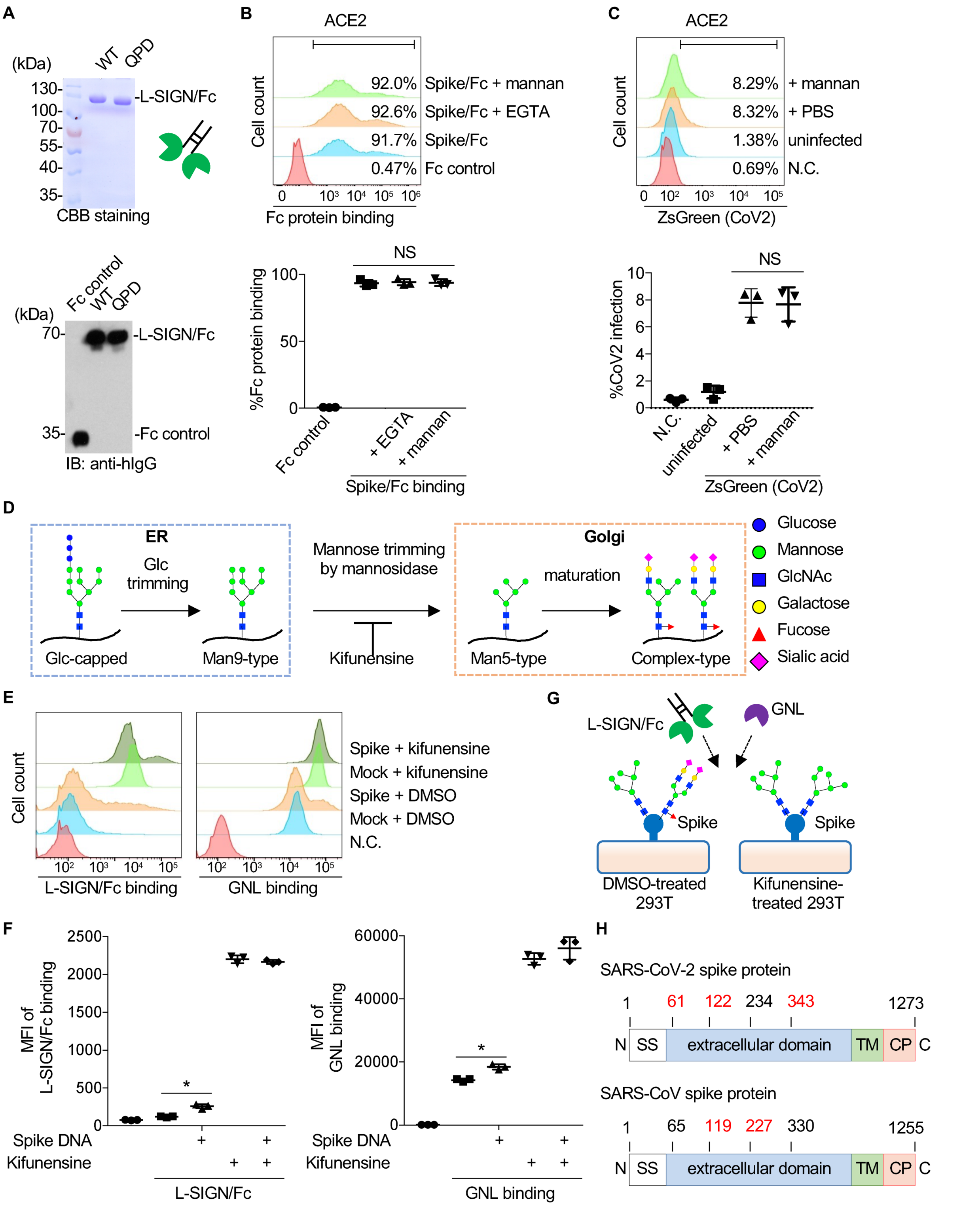


**Supplemental Figure 2. L-SIGN recognizes high-mannose type *N*-glycans on SARS-CoV-2 spike protein**. (**A**) CBB-stained SDS-PAGE gel (top) and western blotting (bottom) of COS-7 cell-derived purified L-SIGN/Fc and L-SIGN$^{QPD}$/Fc proteins. WT, L-SIGN$^{wild\text{-}type}$/Fc; QPD, L-SIGN$^{QPD}$/Fc. (**B**) Representative histogram of flow cytometry of Fc proteins (top) to transfected

ACE2-myc3-expressing HEK293T cells in the presence of EGTA (1 mM) or mannan (100 μg/ml) and quantification of %Fc protein binding (bottom). (**C**) Representative histogram of flow cytometry of CoV2 infection (top) to transfected ACE2-myc3-expressing HEK293T cells in the presence of mannan (100 μg/ml) and quantification of CoV2 infection (bottom). (**D**) Schematic diagram of *N*-glycan biosynthetic pathway. Kifunensine is an inhibitor of mannosidase I, resulting in accumulation of high-mannose type *N*-glycans. (**E**) Representative histograms of flow cytometry of L-SIGN/Fc binding and biotin-labeled GNL binding to spike-protein-transfected HEK293T cells in the presence of kifunensine (10 μM, 48 h). Treatment with DMSO was used as negative control. N.C. is mock transfected cells stained with Fc control or PE-streptavidin only. (**F**) Quantification of mean fluorescence intensity (MFI) of L-SIGN/Fc binding (left) and GNL binding (right) to spike protein-transfected HEK293T cells relative to MFI of negative control in (**E**). (**G**) Cartoon of glycans on cell surface of kifunensine-treated spike-protein-expressing HEK293T cells. (**H**) Comparison of *N*-glycosylation sites of spike protein between SARS-CoV-2 in SARS-CoV. Asn61, Asn122, Asn234, and Asn343 in SARS-CoV-2 spike protein were reported to be attached with high mannose-type *N*-glycans (7, 8). These residues correspond to Asn65, Asn119, Asn227, and Asn330 in SARS-CoV-2 spike protein (9). Numbers are sites in the spike protein sequence. Red numbers indicate reduced infectivity of CoV2 when the site is mutated (See Figure 3F) (10). *$P < 0.05$. For analysis, n = 3. Bars, mean; error bars, s.e.m.

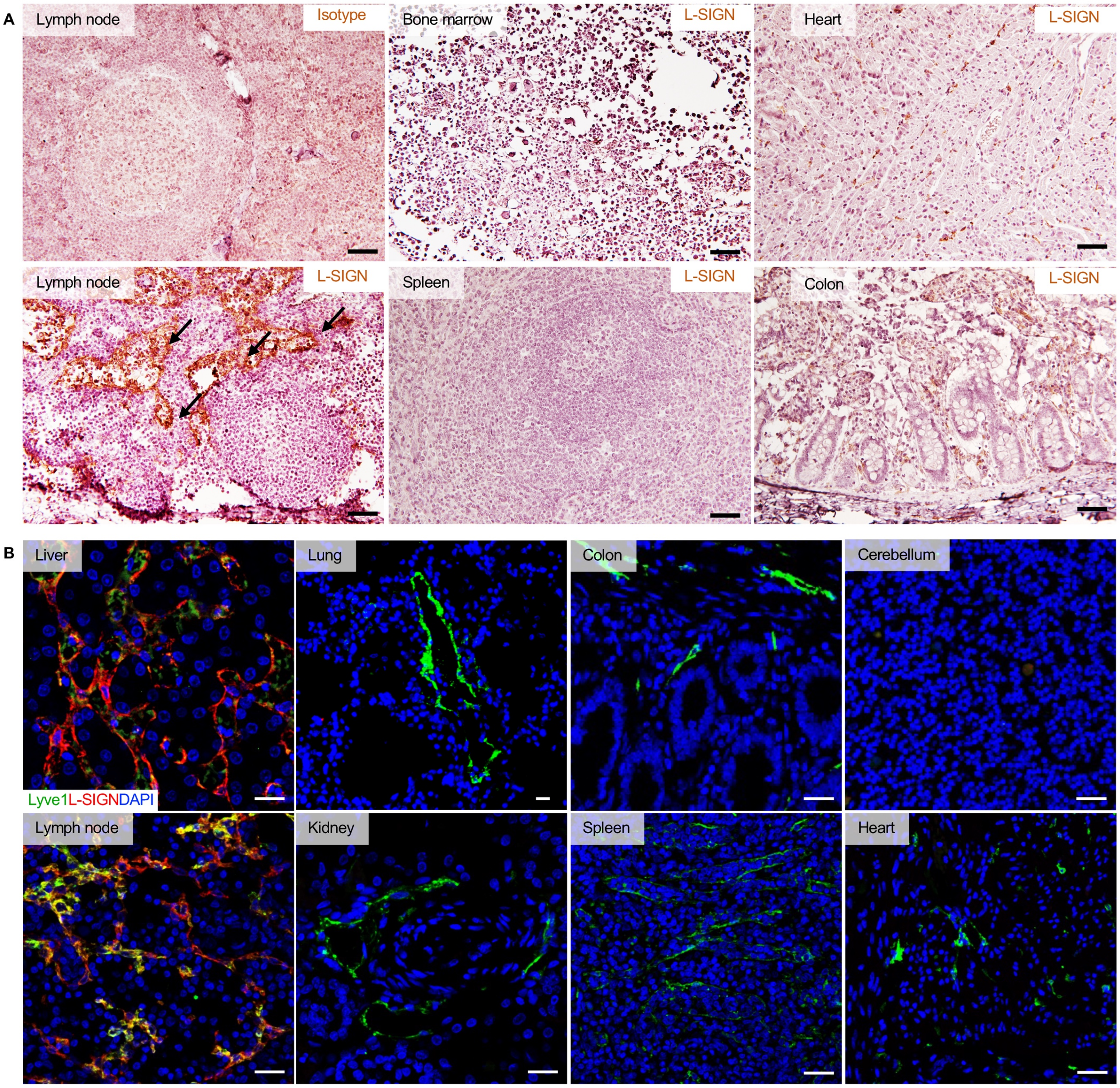


**Supplemental Figure 3. L-SIGN expression profile in different human tissues with validation of L-SIGN expression profile with a different anti-L-SIGN antibody.** (**A**) Images of immunohistochemical stained formalin-fixed paraffin-embedded sections of human autopsy tissue samples. Sections were incubated with mouse anti-human L-SIGN mAb (Origene Cat# TA810067, 1:150). Negative control, isotype-matched control IgG. Scale bar, 25 µm. (**B**) Representative confocal images of immunofluorescent stained formalin-fixed paraffin-embedded sections of human autopsy tissue samples. Lyve1 (green) is LSECs (liver) or LECs (organs other than liver). Unlike prior reports, L-SIGN was not detected in blood endothelial cells (11). Scale bar, 10 µm. Sections were incubated with mouse anti-human L-SIGN monoclonal Ab (ORIGENE Cat# TA810055).

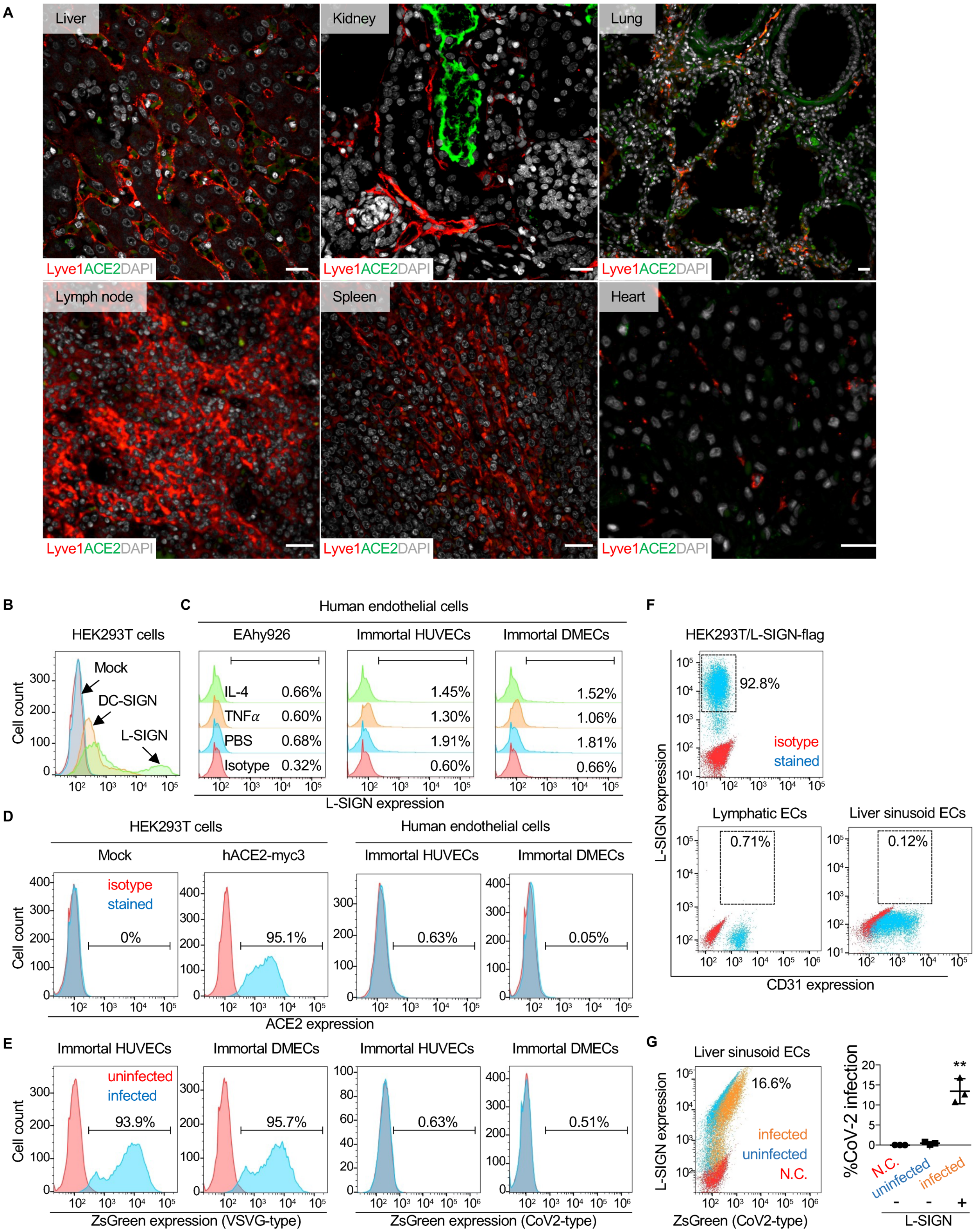
A
Liver
Kidney
Lung
Lymph node
Spleen
Heart
Lyve1ACE2DAPI
B
HEK293T cells
Mock
DC-SIGN
L-SIGN
Cell count
C
Human endothelial cells
EAhy926
Immortal HUVECs
Immortal DMECs
IL-4 0.66%
TNFα 0.60%
PBS 0.68%
Isotype 0.32%
1.45%
1.30%
1.91%
0.60%
1.52%
1.06%
1.81%
0.66%
L-SIGN expression
D
HEK293T cells
Human endothelial cells
Mock
hACE2-myc3
Immortal HUVECs
Immortal DMECs
isotype
stained
0%
95.1%
0.63%
0.05%
ACE2 expression
E
Immortal HUVECs
Immortal DMECs
uninfected
infected
93.9%
95.7%
0.63%
0.51%
ZsGreen expression (VSVG-type)
ZsGreen expression (CoV2-type)
F
HEK293T/L-SIGN-flag
92.8%
isotype
stained
L-SIGN expression
Lymphatic ECs
Liver sinusoid ECs
0.71%
0.12%
CD31 expression
G
Liver sinusoid ECs
16.6%
infected
uninfected
N.C.
ZsGreen (CoV2-type)
%CoV-2 infection
**
L-SIGN

**Supplemental Figure 4. ACE2 is not expressed in LSECs or in lymph node LECs, and L-SIGN and ACE2 are not expressed in cultured endothelial cells.** (**A**) Representative confocal images of immunofluorescent stained formalin-fixed paraffin-embedded sections of human autopsy tissue samples. Lyve1 (red) positive cells are LSECs (liver) and LECs (lymph node), DAPI is nuclear counter staining. Kidney is positive control for ACE2 staining (green). Scale bar, 10 μm. (**B**) Representative flow cytometry histogram showing specificity of anti-L-SIGN mAb. Mock-, L-SIGN-flag, or DC-SIGN-flag-transfected HEK293T cells were stained with mouse anti-human L-SIGN mAb. Isotype-matched control IgG and mock transfected cells were negative controls. (**C**) Representative overlaid flow cytometry histogram of endogenous L-SIGN expression in three different human endothelial cell lines (EAhy926, immortalized HUVECs, and immortalized DMECs) without or with stimulation with TNFα (10 ng/ml) or IL-4 (10 ng/ml) for 24 h. (**D**) Representative flow cytometry histogram of endogenous ACE2 expression in immortalized HUVECs and immortalized DMECs. HEK293T cells stably expressing ACE2-myc3 were positive control. (**E**) Representative flow cytometry histogram of VSVG-type and CoV2-type infection of immortalized HUVECs and immortalized DMECs. (**F**) Representative overlaid dot plots of flow cytometry of endogenous L-SIGN expression in primary liver sinusoid endothelial cells and lymphatic endothelial cells. Cells were co-stained with anti-CD31 antibody. Red, isotype-matched IgG-stained. Blue, anti-L-SIGN antibody-stained cells. HEK293T cells stably expressing L-SIGN-flag were positive control for L-SIGN staining. Dashed boxes in LSECs and LECs indicate infected cells. (**G**) Representative overlaid flow cytometry plots of exogenous L-SIGN expression and CoV2-type infection on L-SIGN-flag transduced LSECs cells (left) and quantification of %CoV2 infection (right). N.C., negative control. Uninfected, L-SIGN-stained. Infected, L-SIGN-stained CoV2-type infected LSECs. For all analyses, n = 3. Bars, mean; error bars, s.e.m. Significance was calculated using a 1-way ANOVA for multiple groups and a 2-tailed Student's t test for comparing 2 groups: *$P < 0.05$; **$P < 0.01$. NS, not significant. All experiments were repeated at least three times.

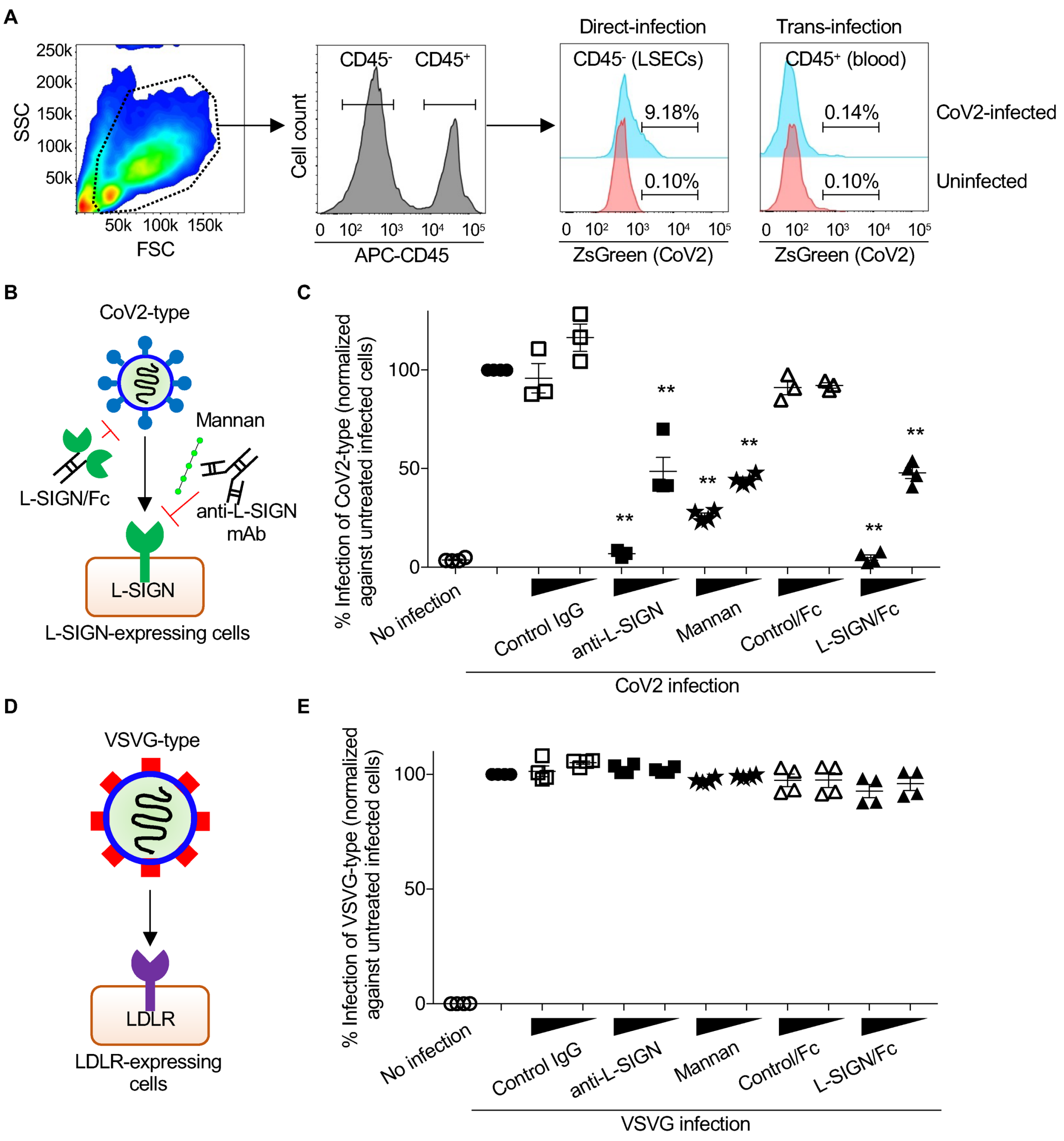


**Supplemental Figure 5. Potential therapeutic option to prevent L-SIGN-mediated SARS-CoV-2 infection.** (**A**) Representative flow cytometry histogram testing L-SIGN-mediated trans-infection in blood cells. L-SIGN-flag-transduced LSECs were pre-incubated with pseudo-typed CoV2 for 2 h, and after being washed, blood mononuclear cells were added. After 48 h, ZsGreen expression in $CD45^+$ blood cells were examined. Uninfected cells were used as negative controls. (**B**) Schematic diagram depicts inhibitory mechanisms of CoV2-type infection in L-SIGN-flag-transfected HEK293T cells by mannan, L-SIGN/Fc, and anti-L-SIGN mAb. (**C**) Inhibition assay of L-SIGN-mediated CoV2-type infection using mouse anti-human L-SIGN mAb (10 and 2 μg/ml), mannan (500 and 100 μg/ml), or recombinant L-SIGN/Fc (50 and 10 μg/ml corresponding to 1 and 0.2 nanomole/ml). Isotype-matched control IgG (10 and 2 μg/ml) and Fc control (1 and 0.2 nanomole/ml) were used as negative control. % Infection efficiency for each inhibitor was normalized against untreated CoV2-infected L-SIGN-expressing cells. (**D**)

Mechanisms of VSVG-type pantropic viral infection. LDLR, low density lipoprotein receptor. (**E**) Inhibition assay of VSVG-type infection in HEK293T cells. % Infection efficiency for each inhibiter was normalized against untreated VSVG-infected HEK293T cells. Mannan, L-SIGN/Fc, and anti-L-SIGN do not block VSVG-type pantropic viral infection. For all analyses, n = 4. Bars, mean; error bars, s.e.m. All experiments were repeated three times.

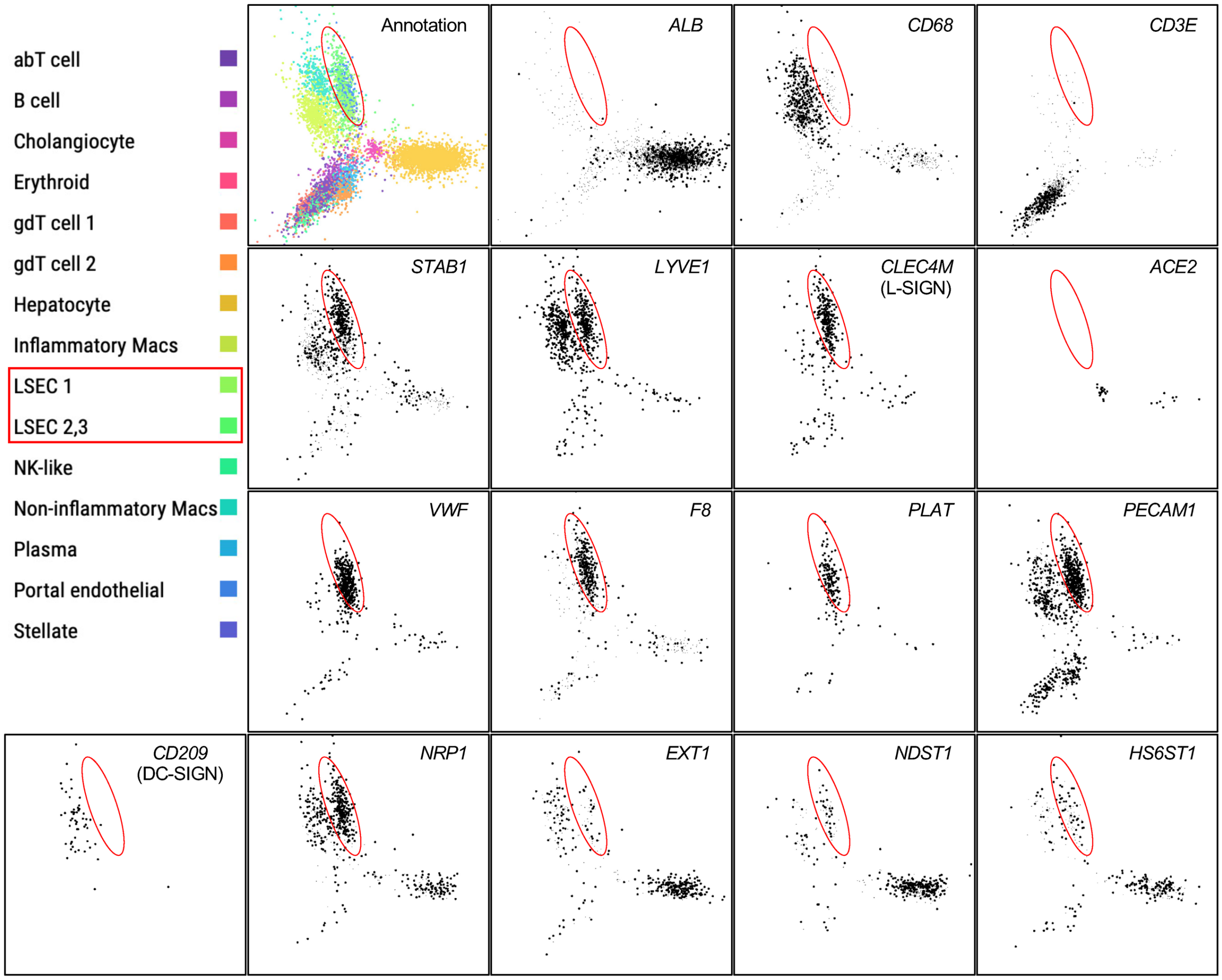


**Supplemental Figure 6. Gene expression profiling of L-SIGN, coagulation factors, and reported SARS-CoV-2 receptors in LSECs.** Co-expression profiles of genes relevant to this study in L-SIGN-positive LSECs, which was extracted from a published database of single cell RNA transcriptional profiles of 8,444 cells from five human liver samples. Similar transcriptome populations were grouped by color-assigned clusters, and LSECs were light green-assigned. Red circles indicate LSEC clusters (annotation). *LYVE1* and *STAB1,* LSEC-specific genes. *ALB* (hepatocyte specific), *CD3E* (T lymphocyte specific), and *CD68* (Kupffer cell-specific) are non-LSEC-specific genes. *PECAM1*, pan-endothelial specific gene. *CD209* is L-SIGN-related gene. L-SIGN and coagulation-related genes (*VWF*, *F8*, and *PLAT*) are highly expressed in LSEC clusters. ACE2 and genes related with HSPG biosynthesis (*EXT1*, *NDST1*, and *HS6ST1*) are negligibly expressed whereas *NRP1* is predominantly expressed in LSECs.

**Reference:**


1. Kondo Y, Fu J, Wang H, Hoover C, McDaniel JM, Steet R, et al. Site-1 protease deficiency causes human skeletal dysplasia due to defective inter-organelle protein trafficking. *JCI Insight.* 2018;3(14).
2. Kondo Y, Ikeda K, Tokuda N, Nishitani C, Ohto U, Akashi-Takamura S, et al. TLR4-MD-2 complex is negatively regulated by an endogenous ligand, globotetraosylceramide. *Proc Natl Acad Sci U S A.* 2013;110(12):4714-9.
3. Herzog BH, Fu J, Wilson SJ, Hess PR, Sen A, McDaniel JM, et al. Podoplanin maintains high endothelial venule integrity by interacting with platelet CLEC-2. *Nature.* 2013;502(7469):105-9.
4. Bergstrom K, Shan X, Casero D, Batushansky A, Lagishetty V, Jacobs JP, et al. Proximal colon-derived O-glycosylated mucus encapsulates and modulates the microbiota. *Science.* 2020;370(6515):467-72.
5. Li Y, Fu J, Ling Y, Yago T, McDaniel JM, Song J, et al. Sialylation on O-glycans protects platelets from clearance by liver Kupffer cells. *Proc Natl Acad Sci U S A.* 2017;114(31):8360-5.
6. Ma X, Li Y, Kondo Y, Shi H, Han J, Jiang Y, et al. Slc35a1 deficiency causes thrombocytopenia due to impaired megakaryocytopoiesis and excessive platelet clearance in the liver. *Haematologica.* 2020.
7. Shajahan A, Supekar NT, Gleinich AS, and Azadi P. Deducing the N- and O-glycosylation profile of the spike protein of novel coronavirus SARS-CoV-2. *Glycobiology.* 2020.
8. Zhao P, Praissman JL, Grant OC, Cai Y, Xiao T, Rosenbalm KE, et al. Virus-Receptor Interactions of Glycosylated SARS-CoV-2 Spike and Human ACE2 Receptor. *Cell Host Microbe.* 2020;28(4):586-601 e6.
9. Walls AC, Park YJ, Tortorici MA, Wall A, McGuire AT, and Veesler D. Structure, Function, and Antigenicity of the SARS-CoV-2 Spike Glycoprotein. *Cell.* 2020;181(2):281-92 e6.
10. Han DP, Lohani M, and Cho MW. Specific asparagine-linked glycosylation sites are critical for DC-SIGN- and L-SIGN-mediated severe acute respiratory syndrome coronavirus entry. *J Virol.* 2007;81(21):12029-39.
11. Jeffers SA, Tusell SM, Gillim-Ross L, Hemmila EM, Achenbach JE, Babcock GJ, et al. CD209L (L-SIGN) is a receptor for severe acute respiratory syndrome coronavirus. *Proc Natl Acad Sci U S A.* 2004;101(44):15748-53.
